# Unveiling the role of *Ndrg1* gene on the oxidative stress induction behind the anticancer potential of styrylquinazoline derivatives

**DOI:** 10.1038/s41598-025-99277-1

**Published:** 2025-05-08

**Authors:** Katarzyna Malarz, Michał Kuczak, Patryk Rurka, Patrycja Rawicka, Anna Boguszewska-Czubara, Josef Jampilek, Jacek Mularski, Robert Musiol, Anna Mrozek-Wilczkiewicz

**Affiliations:** 1 Department of Systems Biology and Engineering, Silesian University of Technology, Akademicka 16, Gliwice, 44-100 Poland; 2 Institute of Physics, University of Silesia in Katowice, 75 Pułku Piechoty 1a, Chorzów, 41-500 Poland; 3 Institute of Chemistry, University of Silesia in Katowice, 75 Pułku Piechoty 1a, Chorzów, 41-500 Poland; 4 Department of Medical Chemistry, Medical University of Lublin, Chodźki 4a, Lublin, 20-093 Poland; 5 Department of Chemical Biology, Palacky University Olomouc, Slechtitelu 27, Olomouc, 779 00 Czech Republic

**Keywords:** Anticancer drug, Styrylquinazoline, Oxidative stress, Iron chelator, *Ndrg1*, p53 protein, Autophagy, Apoptosis, Biochemistry, Cancer, Drug discovery, Molecular biology

## Abstract

This work presents a multifaceted mechanism of the anticancer action of a 2-styrylquinazoline derivative. Extensive analysis of various aspects related to tyrosine kinase inhibition and effects on cellular targets at both the gene and protein levels revealed the potential of this IS20 compound for future research. This study presents a detailed analysis of the relationship between ABL and SRC kinase affecting the inhibition of the EGFR/mTOR signaling pathway in a non-obvious manner. The study was supported by experiments using various molecular biology techniques to confirm the induction of oxidative stress, inhibition of the cell cycle in the G2/M phase and the triggering of cell death via both the apoptosis and autophagy pathways. The cell models included those with different p53 protein status, which affected the cellular response in the form of altered *Ndrg1* expression. Finally, the appropriate physicochemical properties of IS20 for adequate bioavailability and toxicity to the body were observed in an *in vivo* model.

**Supplementary Information:**

The online version contains supplementary material available at 10.1038/s41598-025-99277-1.

## Introduction

**Fig. 1:**
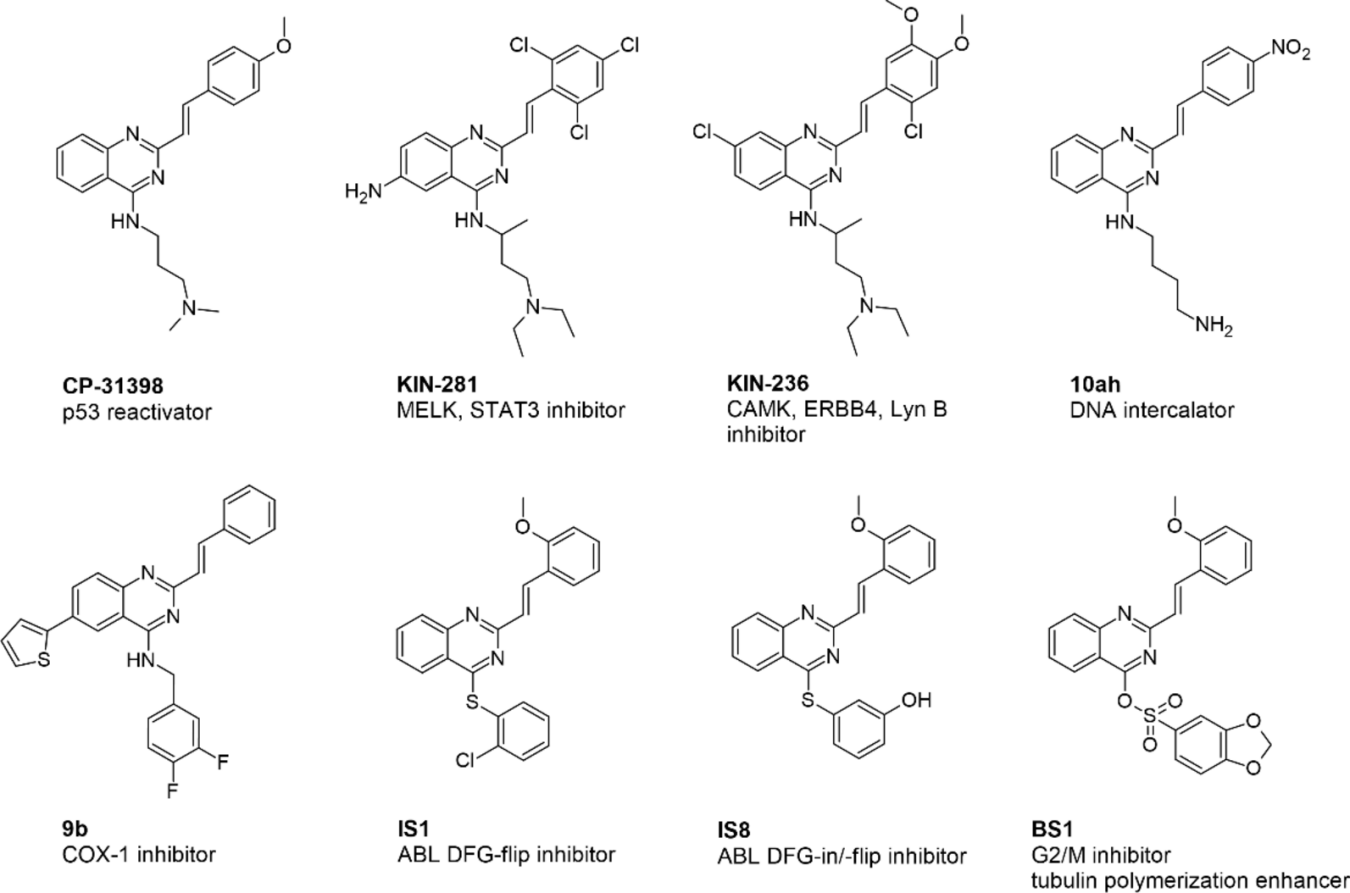
Diversity of 2-styrylquinazoline-core compounds and their biological signature in cancer cells.

**Fig. 2:**
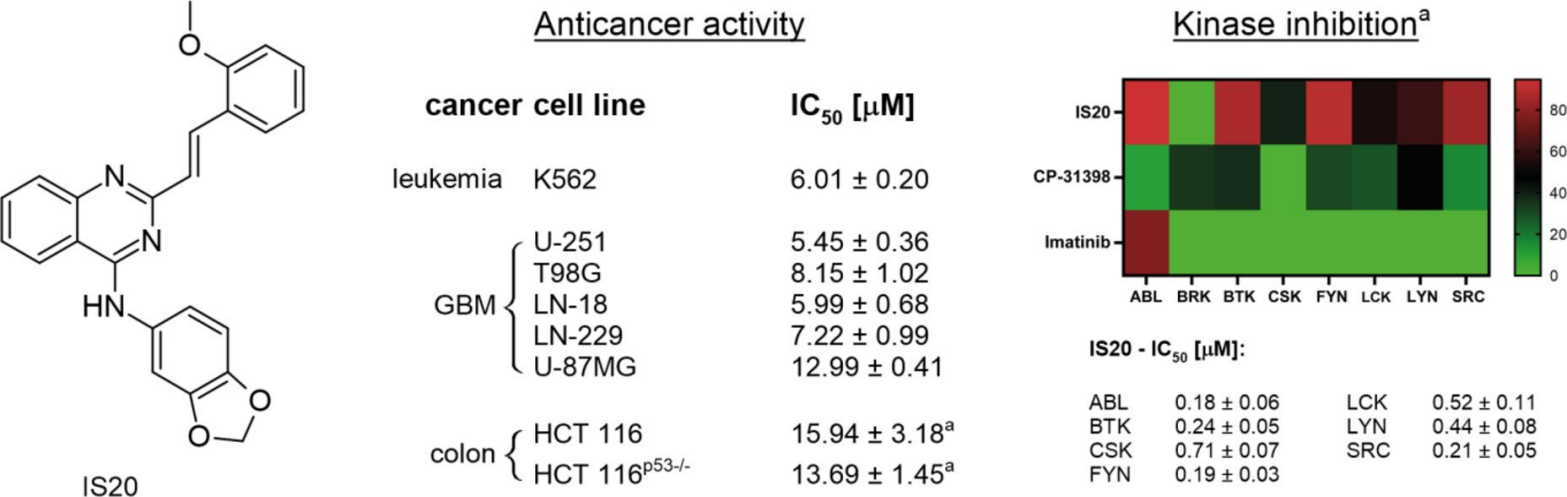
The chemical structure of IS20 (6b) styrylquinazoline, along with its anticancer activity on a panel of cancer cell lines with different *TP53* gene status and kinase inhibition profiles. ^a^Values for anticancer activity of IS20 against HCT 116 cells, as well as kinase inhibition activity by IS20, CP-31398, and imatinib, were taken from our previous work^13^.

The quinazoline scaffold is a versatile and unique motif, which has proved beneficial in drug design and discovery. Compounds containing quinazoline rings have a wide range of biological activities, including therapeutic effects such as antitumor^1–3^, antibacterial^4^, anti-inflammatory^5^, antiviral^6,7^, and antifungal^8,9^. Importantly, this privileged structure is commonly present in many FDA-approved EGFR inhibitors, particularly gefitinib, erlotinib, lapatinib, and afatinib^10^. In recent years, quinazoline compounds containing a styryl moiety have become increasingly popular in medicinal chemistry. It has been possible to identify compounds with interesting antitumor activity, which, despite some common features, are characterized by a diverse molecular mechanism of action (see Fig. 1), some of which show clinical relevance. The first example of a promising compound in the preclinical studies is CP-31398. This smallmolecule is known to be a p53 reactivator and is characterized by both stabilization of the active conformation of wild-type p53 protein and the ability to activate it in some types of cancers harboring a mutation of this protein^11^. Studies on CP-31398 have revealed a more complex mechanism of apoptosis induction in both a p53-dependent and -independent manner via Bax induction^12^. Our team recently identified an interesting enzymatic inhibition profile for CP-31398 against several proteins belonging to the non-receptor tyrosine kinase family^13^. Another structurally very similar compound is KIN-281, described as a MELK, BMX and STAT3 inhibitor^14^. Conversely, its analog KIN-236 can interact with and effectively diminish the activity of a wide range of tumor-related kinase proteins, including the Ca^2+^/calmodulin-dependent protein kinase (CAMK) family, ERBB4 and Lyn B^15^. Diversification and design of novel compounds based on the CP-31398 core led to the discovery and synthesis of a 2-styryl-4-aminoquinazoline (10ah). Its anticancer activity against several cancer cell lines was greater than that of the output p53 reactivator and was shown to be most potent against gastric cancer cells (IC_50_ value below 2 µM). Similarly, the molecular mechanism of its anticancer activity was different and focused on DNA intercalation and upregulation of p53 and p21 to cause G2/M cell cycle arrest, as well as regulation of pro- and anti-apoptotic molecules that led to caspase-dependent apoptosis^16^. Some structural modifications involving the attachment of a thiophene ring can be found in compound 9b, which was an effective and selective COX-1 inhibitor, with possible indications of activity in skin, breast, colorectal and ovarian cancers where COX-1 is overexpressed^17^. Recently, we designed and investigated a series of styrylquinazolines with a thioaryl moiety in the C4 position. Interestingly, we revealed two different binding modes for the IS1 and IS8 derivatives, indicating interaction with the DFG-flip and DFG-flip/DFG-in conformational states of ABL kinase^18^. In turn, the sulfonic styrylquinazoline (BS1) characterized by our team proved to be a potent antimitotic agent that enhanced tubulin polymerization much more than the currently utilized paclitaxel^19^. Notably, our earlier work described the anticancer activity of a series of styrylquinazolines based on a similar structural scaffold to CP-31398. Among them, we indicated molecule 6b (described in this work as IS20, structure in Fig. 2), with a benzodioxol group, could be a promising compound with more than 90% inhibitory potency against non-receptor tyrosine kinases, including ABL, and kinases of the SRC family^13^. It is also worth pointing out that its antiproliferative activity against several types of cancer, was significantly greater than CP-31398.

Despite the great interest in quinazoline derivatives, there are many gaps in fully understanding the action of these compounds on cell biochemistry and interactions with various molecular targets. In addition, the indicated examples show that despite the similar structural pattern, the molecular mechanism of action of the compounds at the cellular level is quite different. Considering this, we decided to explore this area of cellular biochemistry in-depth, focusing on the effect of IS20 derivatives on cellular metabolism, redox disruption, EGFR/mTOR signaling and cell cycle progression in glioblastoma and leukemia cells with altered p53 status. We also examined the mechanism of cell death through both autophagy and apoptosis. Notably, a comprehensive analysis of IS20 action, due to its superior ability to inhibit several tyrosine kinases, may reveal interesting interactions with other molecular targets. Indeed, this could be particularly important in outlining new therapeutic opportunities in designing compounds containing the styrylquinazoline scaffold.

The tumor suppressor protein p53 is a crucial molecule that regulates cell cycle progression, DNA repair, apoptosis, autophagy, and senescence. Under physiological conditions, p53 is typically maintained at low levels, but with increased cellular stress, its levels can upregulate, and it becomes activated, which modulates various cellular events^20,21^. The behavior of p53 may differ under low and high oxidative stress. For example, in response to low levels of oxidative stress, p53 may have an antioxidant function, unlike the occurrence of high oxidative stress, in which p53 exhibits pro-oxidative activities and suppresses antioxidant genes to enhance the level of oxidative status, leading to apoptosis^22^. The frequency of *TP53* gene mutations and p53 protein aberrations is very high in many types of tumors, at approximately 50% of cases. In glioblastoma (GBM), deregulation of p53 signaling occurs in up to 84% of patients. Together with the heterogeneity within the tumor, the multiplicity of genetic abnormalities among the most important signaling pathways (such as EGFR, PI3K, Ras, IDH-1), and the recurrence due to the presence of stem cells makes GBM the most aggressive brain tumor with poor prognosis, and thus difficult to treat^23,24^.

One of the more important downstream targets of p53 is the ABL protein, encoded by the proto-oncogene of the same name, which is involved in cell proliferation, differentiation, and migration. Of note, the *BCR*-*ABL1* fusion transcript is a characteristic lesion of leukemia, but its active form has also been found in some cases of GBM^25,26^. Surprisingly, in recent years, more similarities have been found in leukemic cells among sets of mutations involving signaling pathways and gene expression abnormalities detected in GBM^27^. While p53 has been shown to directly regulate the activity of ABL in response to DNA damage, ABL also interacts with p53, enhancing its transcription and increasing the expression of p21, which can decide cell fate in two ways^28^. Thus, the interplay between p53 and ABL is particularly important for regulating processes leading to cell cycle arrest, suppression of tumor growth and in triggering apoptosis.

## Results and discussion

### Activity landscape of IS20 against cell lines with different *TP53* status

Understanding the molecular mechanism of action of the 2-styrylquinazoline derivative IS20 began by evaluating its antiproliferative profile on five GBM cell lines, including U-251, T98G, LN-18, LN-229, and U87-MG, and a leukemia cell line K562. It is worth noting that both leukemia and GBM cell lines, except U87-MG, were characterized by altered *TP53* expression levels and heterogeneity of p53 mutants. The GBM cells (U-251, T98G, LN-18 and LN-229) carry a point missense mutation resulting in a one amino acid substitution in the codon, which may cause the p53 protein to acquire a gain-of-function feature. Each of these GBM cell lines produces a different version of the final protein due to different amino acid exchange sites in the codons^29^ (Table S1). Conversely, in K562 cells, the p53 protein is inactivated by the loss of one allele and an insertion mutation in exon 5 of the second allele, resulting in the production of a truncated protein of 148 amino acids in length^30^. The loss of wild-type p53 activity is often attributed to the development of chemoresistance. Moreover, altered expression andmutations of this protein result in the acquisition of oncogenic functions, which has a key impact on the regulation of proliferation, growth, survival, transduction and redox signaling in cancer cells^31^.

In general, IS20 exhibited adequate antiproliferative activity levels against cells with p53 mutation. As shown in Fig. 2, the most susceptible cell line to the compound was U-251, for which the calculated IC_50_ value was 5.45 µM. Also, in the case of LN-18 and K562 cells, the biological activity of IS20 was equally high (IC_50_ was around 6 µM). For the remaining GBM cells with a mutation in p53, the IC_50_ value was 7.22 and 8.15 µM. Interestingly, the most resistant cell line was found to be U87-MG, in which the antiproliferative activity of IS20 was reduced more than 2-fold ( IC_50_ 12.99 µM). We have juxtaposed our results with previous data from colon cancer cell lines: HCT 116 p53 wild-type and HCT 116 with p53^−/−^^13^ and found that IS20 was not effective, regardless of the cellular p53 protein status. Such a difference could be due not only to the expression of the *TP53* gene and p53 protein in these lines but also to interactions with other targets and metabolic reprogramming. In addition, the diverse expression landscape present in cells may significantly impact the activity of multi-targeted inhibitors^32^. In this regard, we decided to unravel the mechanism of action of IS20 on two different cell lines, K562 and U-251, with altered p53 status. We further wanted to examine the regulation of metabolism, cell cycle and redox signaling in p53 mutant cells in which the similarity between ABL and IDH-1 enzymes was discovered^32^.

### Destabilization of the cellular redox balance

Maintaining redox homeostasis is an essential process for normal growth, proliferation, survival and regulation of cell signaling pathways. However, if equilibrium is being disrupted, the elevated reactive oxygen species (ROS) drives cellular metabolism, promoting uncontrolled cell growth, tumor progression and metastasis. Hence, cancer cells adapt to increased levels of intrinsic ROS and exhibit an altered capacity of the ROS-scavenging system^33^. Therefore, an approach targeting the disruption of redox homeostasis may be an effective strategy for eradicating cancer cells^34^. Thus, ROS is perceived as a double-edged sword or Trojan horse^35^. It has been shown to facilitate cancer cells’ vulnerability to damage and the effects of oxidative stress due to the excessive increase in ROS concentrations induced by anticancer drugs, and metal chelators^36–39^. It should be noted that excessive ROS can completely deplete the capacity of antioxidant systems, leading to oxidative damage to lipids, proteins, and DNA, and ultimately triggering cell death through apoptosis^35^. In a therapeutic context, modulation of redox equilibrium and the associated cellular signaling is an effective and selective approach due to the significant differences in ROS levels between cancer and normal cells^34^.

**Fig. 3:**
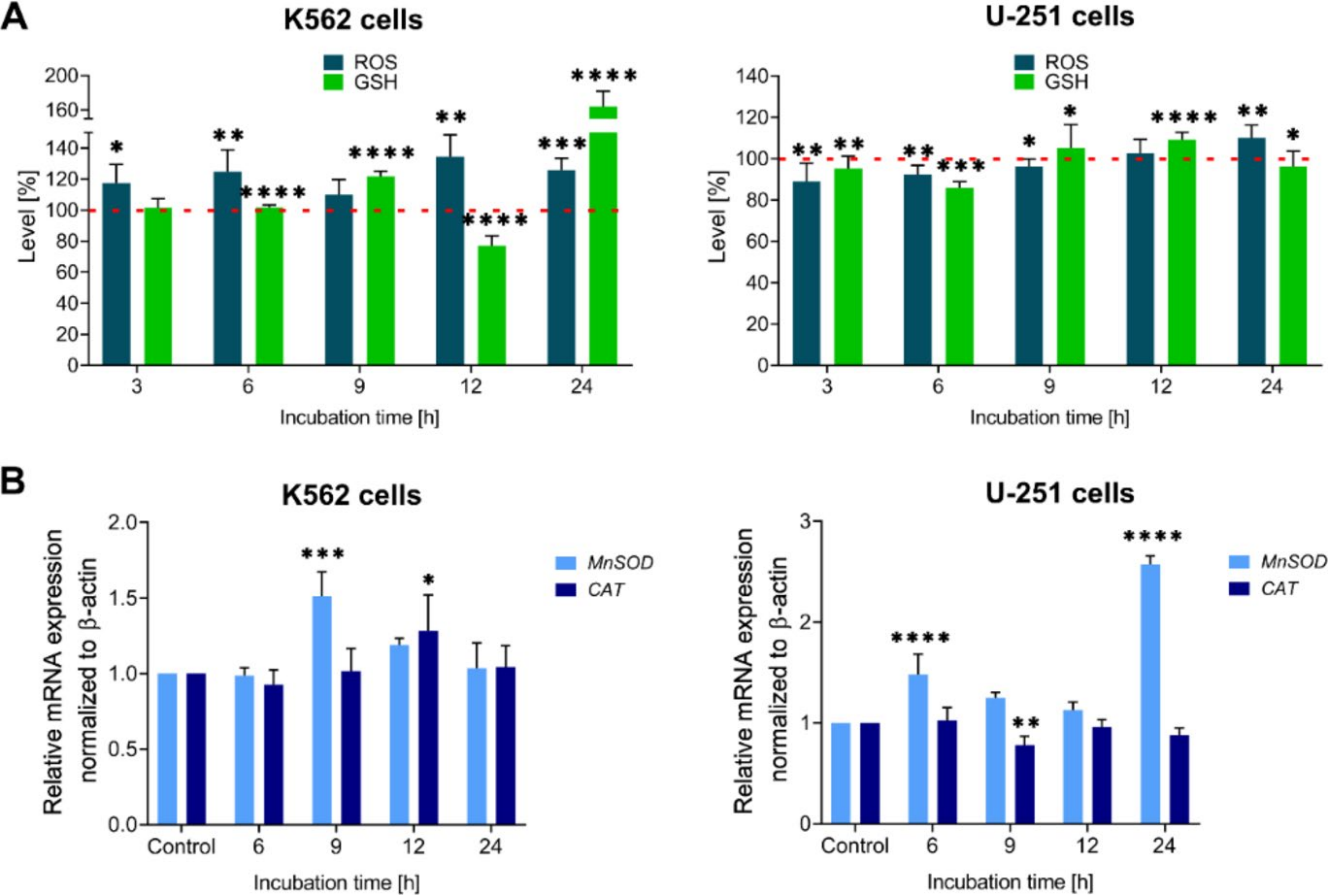
The time-dependent impact of the tested IS20 derivative on ROS (navy blue bars) and GSH (green bars) levels in the K562 and U-251 cells (**A**). The mRNA expression of *MnSOD*, and *CAT* genes in the K562 and U-251 cells after incubation with IS20 in kinetic experiments (**B**). The data were normalized to the control–untreated cells and analyzed using the unpaired t-test: *p < 0.05, **p < 0.01, ***p < 0.001, ****p < 0.0001 compared to the control. The results are presented as the mean ± SD of several independent experiments (*n* = 5). The red dashed lines indicate the standardized levels of ROS and GSH in the control, which is taken to be 100%.

Changes in the level of ROS in leukemia and GBM cells were monitored using a fluorescence CellROX dye assay. Experiments were conducted over 3 to 24 h following treatment with IS20. Our analysis revealed a significant increase in ROS levels in both cell lines (Fig. 3A–navy blue bars), and in particular, a clear trend of growing ROS levels was observed in K562 cells. Elevated ROS levels were observed as early as 3 h following compound administration, after which they continued to increase at subsequent time points, reaching their highest level after 12 h (an increase of more than 135%). Smaller changes were observed in U-251 cells, with the highest level of ROS detected 24 h after exposure to IS20 (over 110%). The observed differences may be due to cellular vulnerability and the ability of the antioxidant system to eliminate ROS. As mentioned above, even small changes in the ROS levels and prolonging this condition can be sufficient to disrupt redox homeostasis, which leads to the induction of oxidative stress. It is noteworthy that the potential of IS20 to generate ROS after 24 h was drastically suppressed after incubation with the antioxidant ascorbic acid (Fig. S1A). Similarly, higher viability of K562 and U-251 cells was observed after incubation with IS20 in combination with ascorbic acid (Fig. S1B). In the literature, it has been shown that CP-31398 can also induce ROS production in cancer cells, which can trigger cell death by apoptosis^40,41^. However, how this p53 reactivator can directly increase ROS levels while also affecting the cellular antioxidant defense system has not been clarified. Moreover, the quinazoline core can exhibit a high affinity for metal ions, which are also present in coordination compounds with transition metals, such as copper^42,43^.

With this in mind, we explored whether 2-styrylquinazoline derivative is capable of chelating ions and forming active redox complexes, as this could explain the disruption of redox homeostasis. Thus, we performed spectroscopic titration of the IS20 solution with iron (III) and copper (II) (Fig. S2). In general, adding both metal ions caused a decrease in absorption intensity at 345 nm. After titration with Fe^3+^, two marked isosbestic points were located at 320 and 370 nm, while for Cu^2+^, only one was observed at 320 nm. These isosbestic points indicate the existence of equilibrium in this region and the occurrence of at least two molecular forms of the compound (free and ion-bound). The isosbestic point located at shorter wavelengths is likely responsible for the electron transitions in the molecule from the ground state to the excited state in the presence of metal ions. In turn, an isosbestic point shifted toward longer wavelengths is associated with the charge transfer mechanism between the ligand and the metal ion^44^. Thus, two well-identified isosbesticpoints may confirm the presence of a stable complex between the IS20 ligand and the Fe^3+^ ion in the solution. In contrast, only one isosbestic point was observed when titrated with Cu^2+^ ions, which may suggest a weak affinity for these ions and a lower chelating ability by IS20.

Next, the response of the antioxidative defense system to the elevation of ROS levels in both cell lines was evaluated. For this purpose, we performed a series of GSH concentration measurements in a similar kinetic manner. We detected many fluctuations in GSH concentration in K562 and U-251 cells (Fig. 3A-green bars), which may indicate that the cellular defense program was activated upon ROS production by IS20. The largest depletion of GSH levels (about 20%) was observed after a 12 h incubation in leukemia cells. It seems that a decrease in antioxidant potential may correlate with the highest ROS concentration. Surprisingly, the GSH levels increased over the following 12 h. Smaller fluctuations were noticed in U-251 cells, in which GSH levels finally decreased after a 24 h incubation with IS20. The reduction of GSH levels can trigger early apoptotic events such as mitochondrial damage followed by cytochrome c release, and a cascade of caspase activation^45^. However, it should be noted, that the GSH system, together with thioredoxin, is not a large detoxification system, but a second-line defense against ROS^46^. Therefore, insight into other antioxidant effectors involved in free-radical leveling is crucial. Antioxidant disruption and loss of potential to nullify ROS may encompass a multifaceted mechanism that involves the regulation of a set of different factors and signaling pathways. Such regulation may result from differential susceptibility to ROS-based therapy.

For this reason, our attention focused on the impact of IS20 on the regulation of superoxide dismutase (*MnSOD*) and catalase (*CAT*) gene expression as elements of the first line of cellular defense, which are responsible for scavenging radicals, such as superoxide (O_2_^−^) or hydrogen peroxide (H_2_O_2_). Gene expression analysis indicated fluctuations in *MnSOD* and *CAT* levels in both cell lines (Fig. 3B). We noticed a significant upregulation of *MnSOD* and *CAT* expression after 9 h and 12 h of treatment with IS20 in K562 cells, respectively. In GBM cells, *MnSOD* levels increased after 6 h and 24 h of treatment, in contrast to the expression of *CAT*, which decreased after 9 h of incubation with IS20. The sustained reduction in *CAT* expression appears to be a response to the prolonged oxidative state, that weakens and depletes resources for nullifying radicals. Moreover, the differences in *MnSOD* responses appear to correspond to a steady increase in ROS levels in both tested cell lines.

### Modulation of oxidative stress-related and iron-regulated proteins

**Fig. 4:**
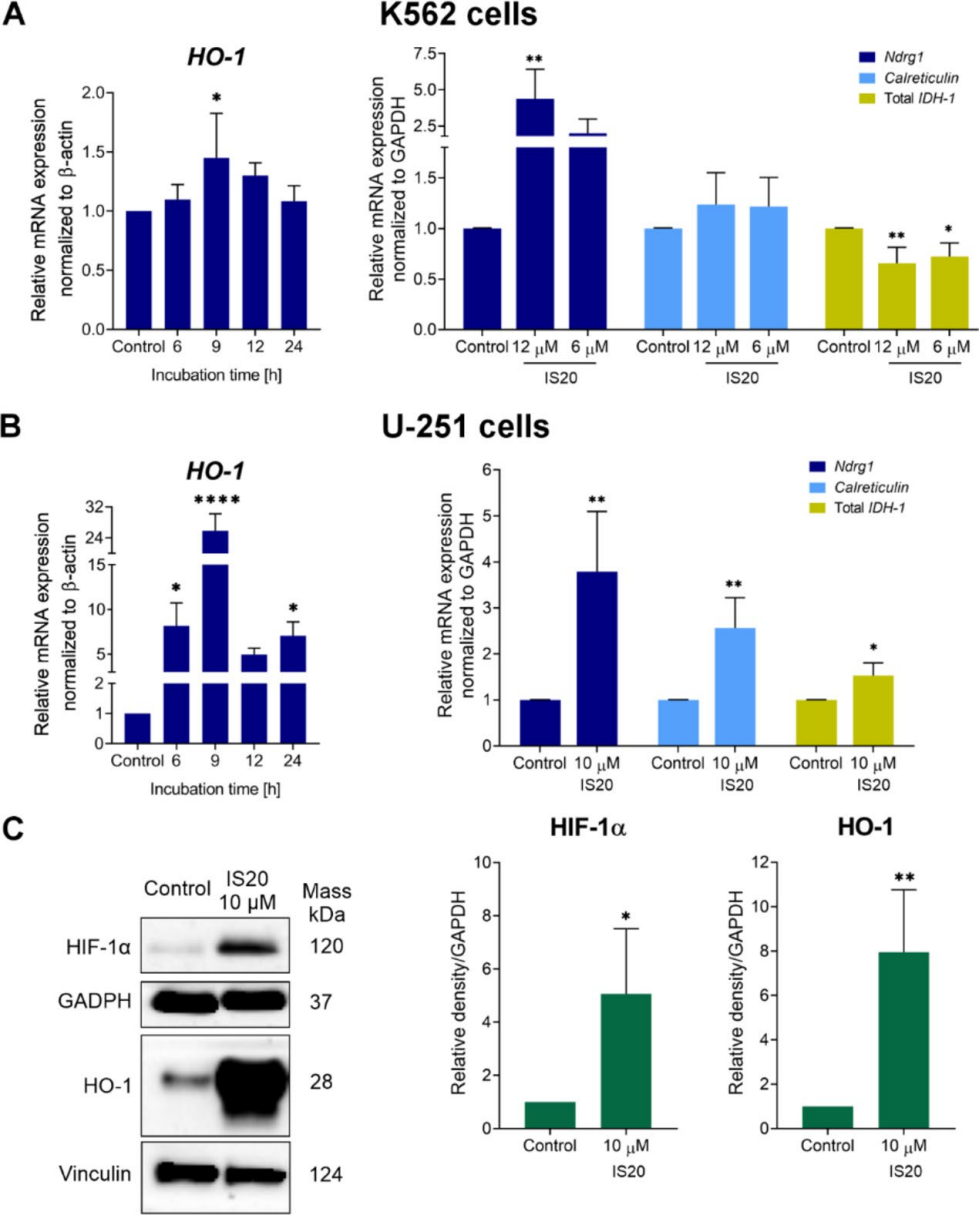
The impact of IS20 on the expression of selected genes and proteins associated with oxidative stress and iron regulation in the K562 (**A**) and U-251 cells (**B**,**C**). The *HO-1* expression was determined in kinetic experiments, while *Ndrg1*, *calreticulin*, and *IDH-1* gene expression after 24-hour incubation. The levels of HO-1 and HIF-1α proteins were determined after 24-h treatment with IS20 in U-251 cells. The results of gene and protein expression are presented as the mean ± SD of several independent experiments (*n* = 5). Immunoblots shown are representative of five independent experiments. The displayed blots were cropped, and the full-length blots are shown in Fig. S6. The statistical analysis was performed using a t-test or one-way ANOVA with Bonferroni’s posthoc test: *p < 0.05, **p < 0.01, ***p < 0.001, ****p < 0.0001 compared to the untreated controls.

To comprehensively assess the induction of oxidative stress by IS20, we examined changes in heme oxygenase-1 (HO-1) at the gene and protein levels. This molecule has a pro- and antioxidant role in cells depending on the status of redox homeostasis and the fate of iron distribution^47^. HO-1 is responsible for heme degradation to biliverdin, releasing carbon monoxide and ferrous iron (Fe^2+^). Biliverdin and its transformation product, bilirubin, can act as powerful antioxidants. However, increased HO-1 expression may lead to an enrichment of iron availability in the mitochondrial pool^48,49^, thereby increasing iron accumulation and inducing cell damage^50^. We found an almost 1.5-fold increase in *HO-1* gene expression was registered in K562 cells after a 9 h incubation with IS20 (Fig. 4A). Conversely, remarkable changes were found in U-251, where *HO-1* gene expression increased at least more than 5-fold during the entire incubation (Fig.  4B). Of note, a strong, more than 25-fold, increase in *HO-1* levels was recorded in a 9 h incubation with IS20. At the protein level, we also confirmed significant upregulation of HO-1 expression in U-251 cells (Fig.  4C). It should be noted that another protein associated with the destabilization of redox balance, and an increase of oxygen levels in the cellular environment is HIF-1α. Therefore, it is not surprising that IS20 caused a 5-fold increase in HIF-1α protein levels in the GBM cells. Unfortunately, both mentioned proteins were undetectable in leukemia cells using the Western Blot technique.

Another crucial molecule in the cellular stress response is the iron-regulated metastasis suppressor N-myc downstream-regulated gene 1 (Ndrg1). This molecule negatively regulates cancer cell growth, differentiation, invasion and migration^51^. It is also a powerful target for anticancer therapy due to its role in many significant cellular processes, and its reduced expression is found in several types of cancer, such as brain, breast, and pancreatic^51,52^. For example, Sun et al. revealed that decreased *Ndrg1* gene expression in gliomas could contribute to carcinogenesis, cancer progression and survival^53^. Most importantly, several studies by Richardson’s group have shown that Ndrg1 expression can be regulated by factors that induce hypoxia, DNA damage and changes in intracellular iron^54^. Indeed, the iron chelators from the thiosemicarbazone group (i.e., Dp44mT), as well as deferoxamine (DFO), can induce strong upregulation of Ndrg1 expression at the gene and protein levels by decreasing iron levels in cells^55,56^. Moreover, Ndrg1 expression can be augmented by HIF-1α–dependent and –independent mechanisms^56^. The qRT-PCR analysis showed that *Ndrg1* gene expression was strongly increased following treatment with IS20 in both cell lines (Fig.  4 A, B), with a more than 3.7-fold upregulation of *Ndrg1* in the U-251 cell line. Notably, the effect was slightly stronger in K562 cells. Moreover, this regulatory mechanism appears to depend on HIF-1α, which was elevated in GBM cells. Interestingly, both molecules may also promote endoplasmic reticulum stress (ER stress), which can exist and crosstalk with oxidative stress^57,58^. Elevated ROS levels can deregulate redox homeostasis at several sites in cells, such as the mitochondria, cytosol and ER. The ER is responsible for protein synthesis, folding, post-translationalmodifications, as well as maintaining calcium homeostasis. However, it is sensitive to any changes in the cellular environment. Thus, if the antioxidant defense is ineffective, several cellular events occur that have multilevel effects on the regulation of signaling pathways, including Ca^2+^ influx, energy demand, hypoxia, ER stress, or autophagy^57^. These findings prompted us to investigate the impact of IS20 on the *calreticulin* expression, a gatekeeper of the Ca^2+^ pool in the ER. Indeed, we detected a greater than 2.5-fold increase in the expression of this gene in GBM cells (Fig.  4B). This is consistent with previous data, in which Waser et al. suggested that depletion of intracellular Ca^2+^ stores in the ER induces *calreticulin* gene activation in both *in vitro* and *in vivo* models^59^. Moreover, recent reports have shown that overexpression of calreticulin can enhance autophagosome formation and autophagy-related cell death under ER stress^60^. The mechanism of this activation focused on interactions with LC3 protein through the LC3-interacting region (LIR) motif conserved in the calreticulin protein^60^. Metal chelator studies demonstrated enhanced Ndrg1, calreticulin and LC3-II expression^58^. Yet, surprisingly, detailed analysis showed that endogenous overexpression of Ndrg1 in cells may act as a suppressor of autophagy and an enhancer of apoptosis^61^. Interestingly, no significant changes in *calreticulin* expression were observed in leukemia cells. These data reinforce our belief that the two types of p53-mutated cancers have different susceptibility to IS20-induced oxidative stress. The indicated changes are also of interest due to the impact of styrylquinazoline on induced cell death through pathways related to both autophagy and apoptosis.

Furthermore, we evaluated the impact of IS20 on cellular metabolism under stress conditions by determining *IDH-1* expression in both tested cell lines. The IDH-1 protein is involved in the tricarboxylic acid (TCA) cycle, one of the most essential cellular reactions. During the TCA cycle, reduced nicotinamide adenine dinucleotide phosphate (NADPH) co-enzyme is produced, which is essential for cellular processes that maintain redox balance and facilitate the production of lipids and nucleotides^62^. Notably, a reduction in NADPH availability has implications for the induction of oxidative stress due to the depletion of the GSH pool and the thioredoxin system^63^. Shi et al. have indicated that the overexpression of IDH-1-R132H in glioma cells caused a decrease in NADPH levels, resulting in an increase in ROS levels and a decrease in GSH levels^64^. Our analysis indicated that IS20 applied at a dose of 10 µM caused a significant, almost 1.5-fold increase in the *IDH-1* levels in U-251 cells (Fig.  4B). Notably, gene analysis showed the total mRNA expression, while IDH1 protein is often mutated in GBM cells at the arginine site at position 132. Importantly, the opposite effect was registered in leukemia cells, where an almost 1.5-fold downregulation was detected (Fig.  4A). Considering the literature, this change is favourable and may lead to an increase in total ROS levels and oxidative stress. Interestingly, the different behaviour of IS20 in these cellular environments may be related to stimulating alternative pathways due to different basal levels of *IDH-1* in both tested cell lines, as well as existing mutant forms of IDH1 protein^32^.

### Inhibition of EGFR/mTOR signaling pathway

**Fig. 5:**
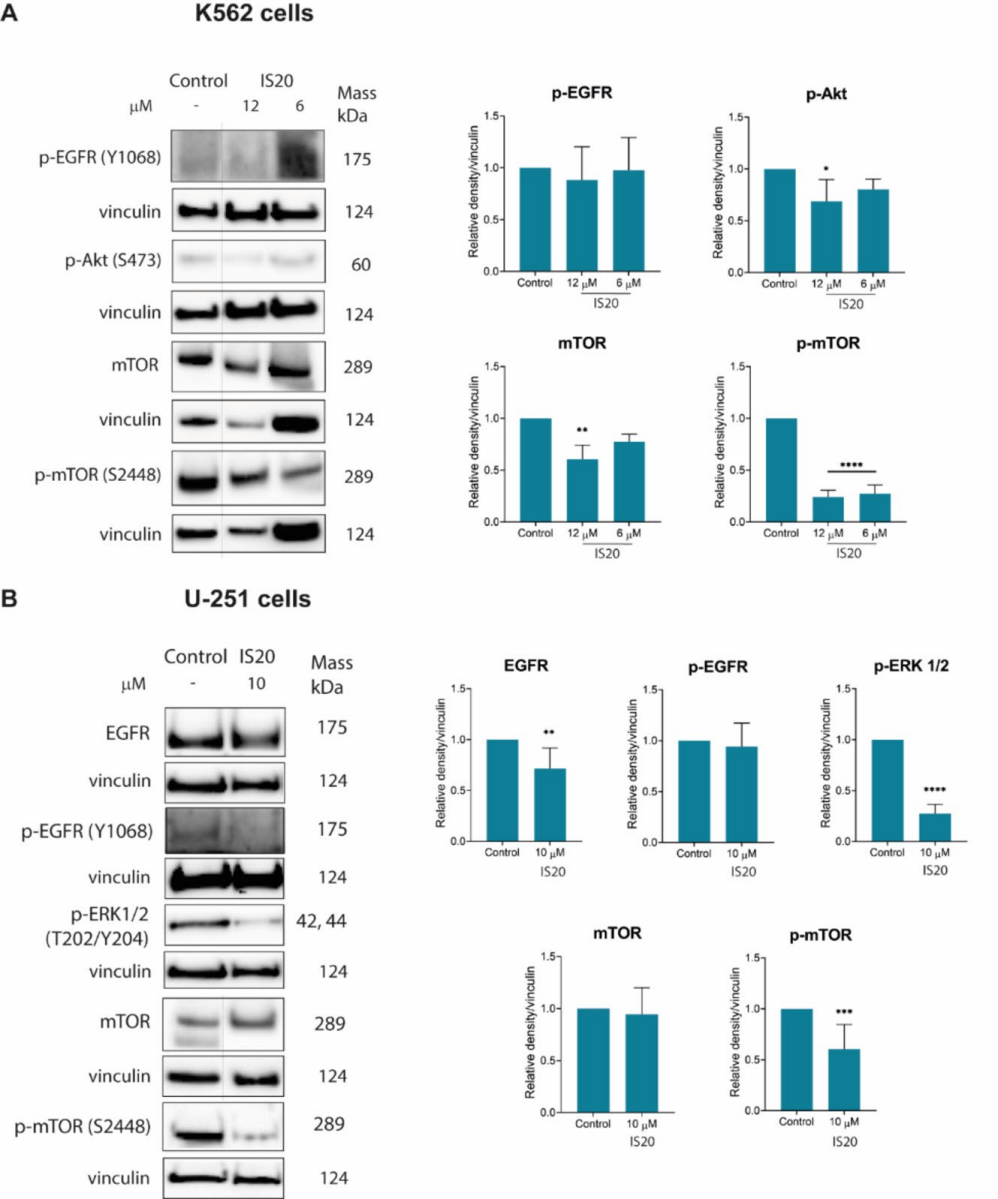
The impact of IS20 on the expression of EGFR and its downstream signaling proteins in K562 (**A**) and U-251 (**B**) cells. The results of protein expression are presented as the mean ± SD of six independent experiments (*n* = 6). Immunoblots shown are representatives from these independent experiments. The displayed blots were cropped, and the full-length blots are shown in Fig. S6. Statistical significance is presented above as *p < 0.05, **p < 0.01, ***p < 0.001, ****p < 0.0001 relative to the respective control, statistical analysis was done using one-way ANOVA with Bonferroni’s posthoc test (for Western Blot) or Dunn–Šidák correction test (for Lumit Immunoassay).

As mentioned above, we showed that the IS20 compound can inhibit several non-receptor tyrosine kinases, such as ABL and SRC. We then checked the possibility of blocking receptor tyrosine kinases from the ErbB and insulin-related families. As depicted in Table S2, the styrylquinazoline at 1 µM concentration demonstrated moderate potential to inhibit the activity of EGFR and HER2 receptor kinase (approximately 25 to 30% inhibition of activity). We registered slightly higher values for insulin-like growth factor 1 receptor (IGF1R), whose activity was blocked by 40% following IS20 administration. Nevertheless, we also examined the possibility of inhibiting the EGFR signaling pathway with its downstream targets at the cellular level. Our assumptions about the impact of the tested compound on this signaling pathway were based on the knowledge that Ndrg1 can interact with and suppress many oncogenic signaling pathways by downregulating EGFR expression^65^. An important example of this may be the inhibition of SRC kinase activation through upregulation of Ndrg1 and interaction with EGFR, which may translate into downregulation of signaling pathways like p130^Cas^ substrate and Abl^66^. For this purpose, we determined the expression of EGFR, phosho-EGFR at Tyr1068, phospho-Akt at Ser473, phospho-ERK1/2 (p44/42) at Thr202 and Tyr204, mTOR and p-mTOR at Ser2448 after exposure to IS20 using the Western Blot method. We found that IS20 caused a significant reduction in the EGFR activation in GBM cells (Fig. 5). In addition, some fluctuations in the phosphorylation of EGFR protein in both cell lines were observed. It appears that IS20 caused a slight decrease in the expression of EGFR phosphorylated at Tyr1068. However, to clarify, we performed additional experiments to determine the p-EGFR levels using the Lumit Immunoassay, which gives more linear read-outs for the level of protein phosphorylation, making it more suitable to discover more minute changes (Fig. S3)^67^. The data from this assay showed that IS20 caused a significant, greater than 1.5-fold decrease in p-EGFR levels in both K562 and U-251 cells. These findings suggest that IS20 may interfere with EGFR activation and phosphorylation, which may affect the transduction of the signaling cascade in cells. Moreover, further analysis of Western Blot results confirmed EGFR pathway inhibition through reducing levels of downstream targets, such as p-Akt and p-ERK1/2. Moreover, IS20 at 12 µM caused a significant, almost 1.5-fold decrease in the expression of Akt phosphorylated at Ser473 in leukemia cells. It should be noted that this protein is a crucial effector of cell proliferation, metabolism, survival and migration. In addition, literature revealed that overexpression of Ndrg1 can regulate PI3K/Akt signaling and GSK3ß protein in GBM cells, resulting in inhibition of cell proliferation and invasion^68,69^. Similarly, the ERK1/2 protein, which belongs to the MAPK protein family, regulates cancer growth and proliferation. By upregulating Ndrg1, iron chelators may deactivate the MEK/ERK signaling cascade, that is stimulated by EGFR^65^. Hence, we detected a significant, greater than 3.6-fold downregulation of p-ERK1/2 after exposure to IS20 in GBM cells. Unfortunately, the level of this protein in leukemia cells was undetectable.

Finally, we evaluated the expression of mTOR signaling after incubation with IS20. The mTOR protein regulates metabolism, activates cell survival, and suppresses autophagy. Thus, insights into the altered expression of this protein may be important given the stimulation of ER stress by IS20 through the calreticulin activation, which affects autophagosome formation. Generally, expression of mTOR phosphorylated at Ser2448 was inhibited after exposure to IS20 in both cell lines. The increased effect was observed in K562 cells, in which IS20 caused a significant 3.5-fold decrease in the expression, while we detected an almost 1.5-fold reduction in the expression of p-mTOR levels in GBM cells. These results were also confirmed by Lumit immunoassay, as depicted in Fig. S3. In summary, these data suggest that IS20 can induce autophagy as a result of oxidative and ER stress occurring in the cells. To confirm this hypothesis, we further verified the induction of autophagy using flow cytometry and determined the expression of two autophagy-related genes (*LC3* and *p62*).

### Cell cycle arrest and regulation of cell cycle-related proteins

**Fig. 6:**
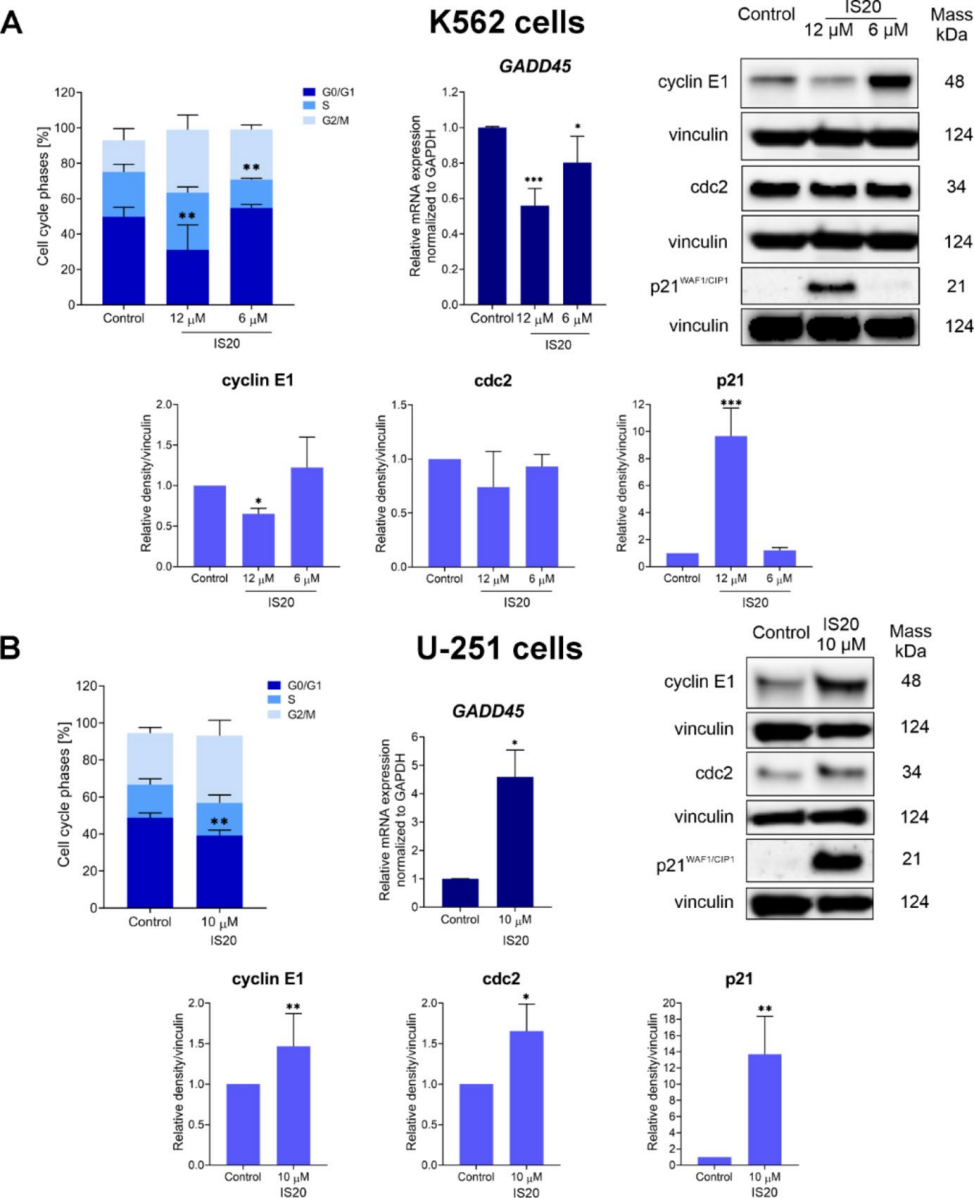
The impact of IS20 on the progression of the cell cycle and related molecules in the K562 (**A**) and U-251 (**B**) cells. The chart presents the percentage of cells in the G0/G1, S and the G2/M phases of the cell cycle. The mRNA expression of *GADD45* in the cells and immunoblotting results with densitometric analysis of cyclin E1, cdc2 and p21 ^CIP1/WAF1^ protein expression after 24-hour treatment with IS20. Results were normalized to the reference gene/protein and are from five independent experiments (*n* = 5). Immunoblots shown are representative from these independent experiments. The displayed blots were cropped, and the full-length blots are shown in Fig. S6. All presented data were analyzed using a one-way ANOVA with Bonferroni’s post-hoc test: *p < 0.05, **p < 0.01, ***p < 0.001, ****p < 0.0001 compared to the control.

To more deeply evaluate IS20, we determined its effect on the cell cycle progression and the change in expression of cell cycle-related genes and proteins. The earlier described changes regarding redox disturbance and iron homeostasis can result in cell cycle inhibition and apoptosis induction. Therefore, we performed a series of flow cytometry, qRT-PCR, and Western Blot experiments on K562 and U-251 cells (Fig. 6). Analysis of the flow cytometry data showed that IS20 causes a significant increase in the cell population in the G2/M phase in both cell lines. Simultaneously, the cell number in the G0/G1 and S phases is reduced. Detailed histograms and a table with the percentage of cells in each phase of the cell cycle are shown in Fig. S4. These findings indicate cell cycle arrest in the G2/M phase by IS20. Despite the similar inhibition of the cell cycle induced by IS20 on both cancer cells, interesting changes were noticed at the molecular level, including a significant 1.8-fold decrease in the expression of *GADD45* after exposure to IS20 at 12 µM in K562 cells (Fig. 6A). The opposite effect was registered in GBM cells, in which a more than 4.5-fold rise in *GADD45* mRNA levels was observed (Fig. 6B). Interestingly, the similar trend was observed in our earlier studies on sulfonic styrylquinazolines^19^. *GADD45* plays a crucial role in G2/M checkpoints, and its stimulation is often associated with DNA damage and stress signal responses, causing cell cycle arrest and the induction of apoptosis. Notably, this gene can be regulated by both p53-dependent and p53-independent pathways. In addition, upregulation of *GADD45* can recruit and activate p38 and JNK kinases, which can mediate the oxidative stress response depending on the potency of the effector/ligand^70^. In the context of cell death induction, *GADD45* may also act together with *CDKN1A* encodes p21^CIP1/WAF1^ protein, and our analysis indicated that IS20 caused significant activation of p21^CIP1/WAF1^ in both cell lines. A greater effect was observed in GBM cells, where we noted an almost 14-fold increase in the level of this protein. These data are consistent with our previous reports on iron chelators, in which the activation of the protein was associated with early hallmarks of apoptosis^36,39^. Moreover, it was observed that p21 was an important stimulator of different cellular responses concerning the destabilization of redox homeostasis, which correlated with different cellular susceptibility to changes in ROS concentrations^39^. It is worth mentioning that Ndrg1 can also upregulate p21 through transcriptional and post-transcriptional mechanisms^71^. In the case of styrylquinazolines, Zhong et al. found that CP-31398 arrested the cell cycle in the G2/M phase, which can be assigned to the enhancement of the p21 protein^41^. Similarly, KIN-281 increased p21 expression in a p53-dependent manner, contributing to cell growth inhibition^14^.

Other proteins examined were cyclin E1 and cdc2, which are responsible for the transition from G0/G1 to S phase and from G2 to mitosis of the cell cycle, respectively. Interestingly, we observed a different pattern of IS20 action on tested cancer cell lines. The cyclin E1 and cdc2 were slightly downregulated after exposure to IS20 at 12 µM in the K562 cells (Fig. 6A), while in U-251 cells, the opposite effect was observed where IS20 induced a more than 1.5-fold increase in these proteins (Fig. 6B). An explanation for the different activation of these cell cycle proteins may be the involvement of p53 in the network of interactions and responses to the breakdown of cellular homeostasis and the induction of multilineage oxidative stress. Indeed, the leukemia cells studied were devoid of p53 protein, while the GBM cells harbored a mutated form of it.

### Dual cell death activation by autophagy and apoptosis

**Fig. 7:**
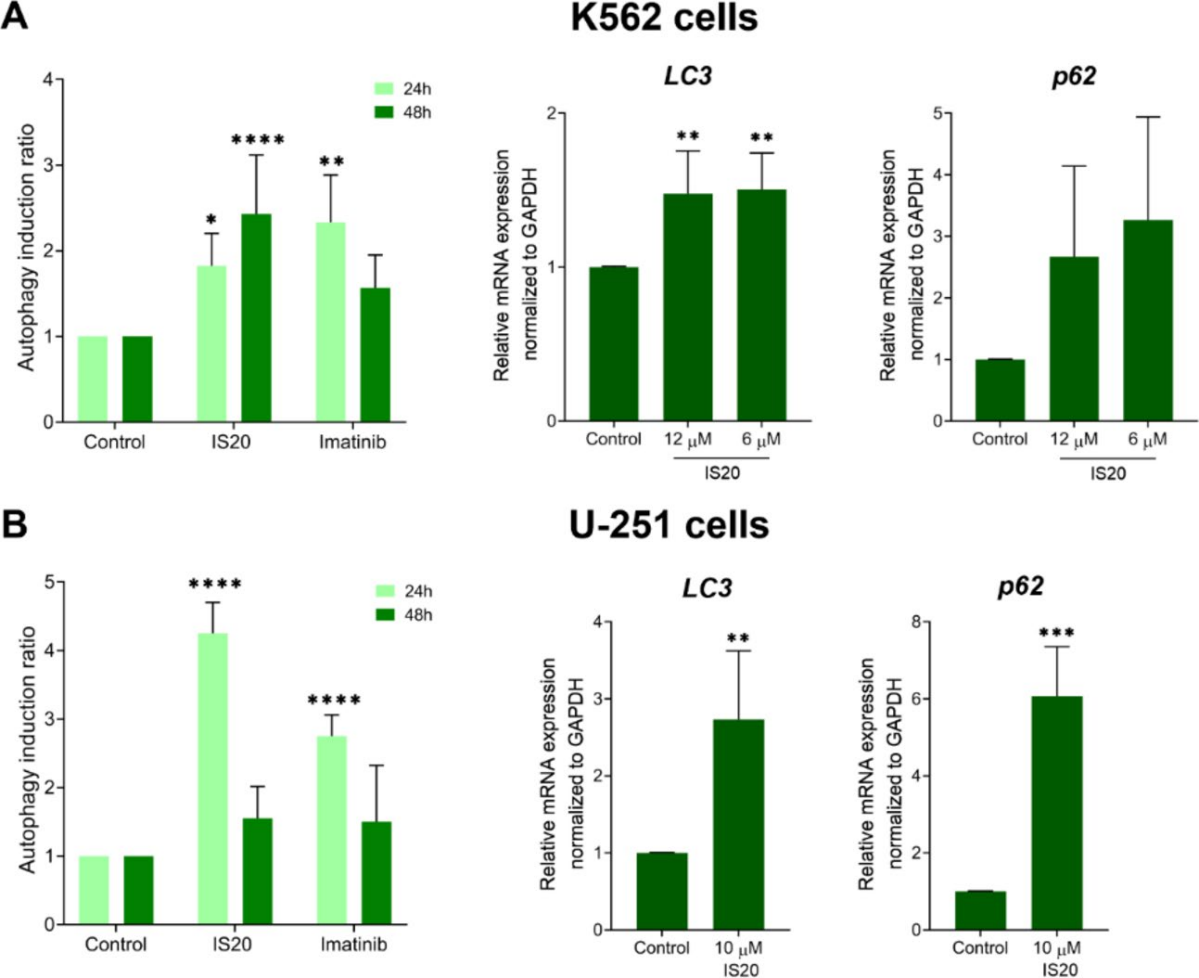
The influence of IS20 on the autophagy induction and expression of autophagy-related genes in the K562 (**A**) and U-251 (**B**) cells. The expression of *LC3* and *p62* genes was determined in both cell lines after a 24 h incubation with IS20. The results are presented as the mean ± SD from five independent experiments (*n* = 5). The statistical analyses were carried out using a one-way ANOVA with Bonferroni’s post-hoc test: *p < 0.05, **p < 0.01, ***p < 0.001, ****p < 0.0001 compared to the untreated cells (control).

To summarize and verify previously obtained results, we conducted experiments to determine the type of cell death induced by IS20. For this purpose, we conducted an experiment using flow cytometry, measuring the fluorescence of an anti-LC3 mouse monoclonal antibody conjugated to Alexa Fluor 555 (Fig. 7 and Fig. S5). This assay allows quantitative measurement of autophagy level. These results indicate a significant increase in autophagy induction ratios in both tested cell lines following incubation with IS20. The largest increase of more than 4 times was recorded for the U-251 cell line after just 24 h of incubation (Fig. 7B). A 2.5-fold increase was registered for the K562 cell line, however, this occurred after a longer 48-hour incubation time (Fig. 7A). Notably, these levels are higher than those of the reference Imatinib. In addition, experiments were performed using qRT-PCR to determine the levels of *LC3* and *p62* genes, which are considered markers of autophagy^72,73^. For *LC3*, the increase in mRNA levels was greatest for the U-251 cell line (more than 2.5-fold), while for the leukemia cells, it increased 1.5-fold. Significant increases in the *p62* gene were registered for both cell lines, with the largest (6-fold) for glioblastoma and about 3-fold for the leukemia cells. The elevated level of both genes indicates the presence of autophagy. These results are consistent with those obtained previously and demonstrate that iron binding by chelators increases theexpression of Ndrg1, which in turn affects the activation of HIF-1α^54^. Another example is the interrelation of autophagy with an increase in calreticulin^60^, the occurrence of oxidative stress^74^ and inhibition of the mTOR pathway^75^.

**Fig. 8:**
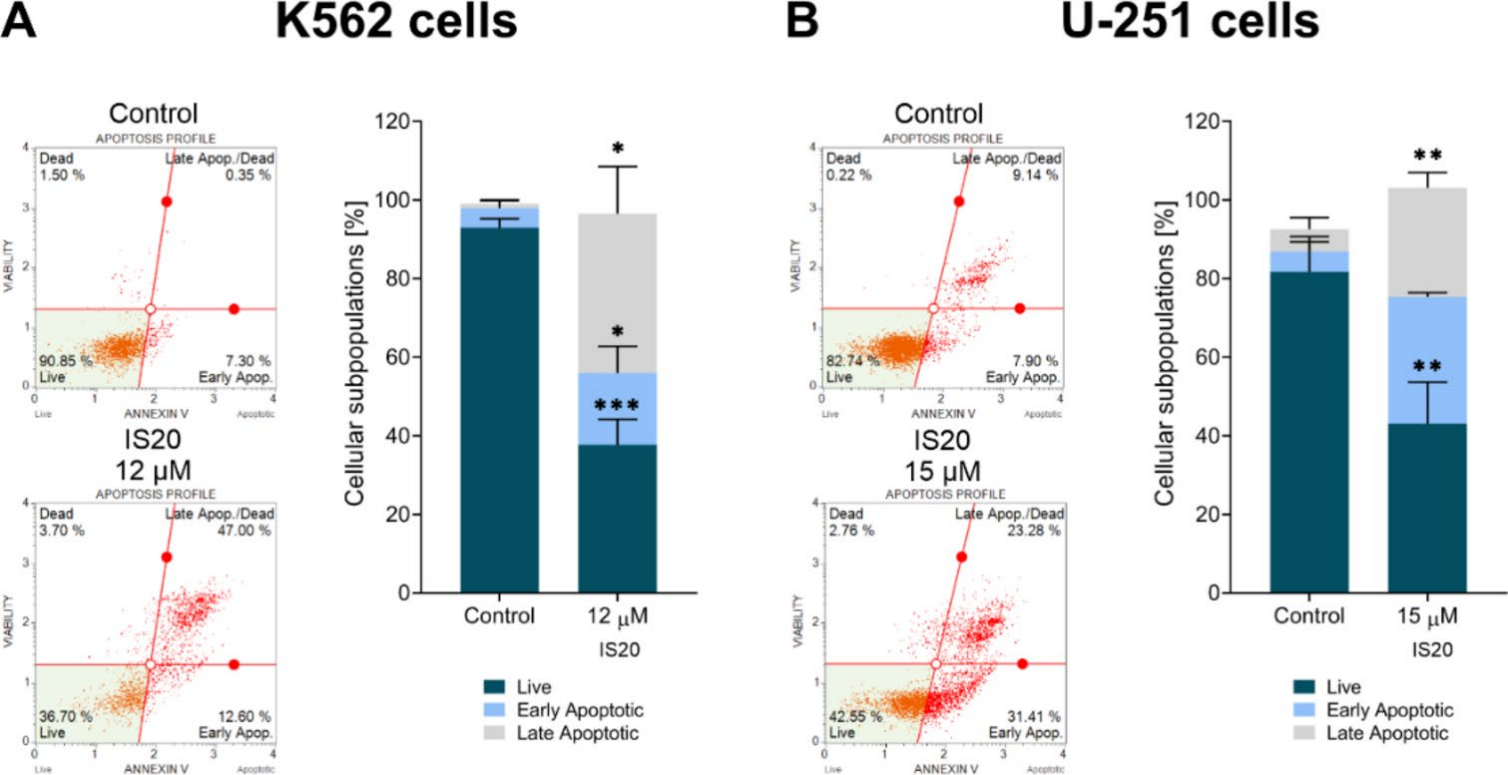
The impact of IS20 on the induction of apoptosis in the K562 (**A**) and U-251 (**B**) cells. The histograms display the percentage of live, early, and late independent experiments. The statistical analysis of the data was performed using a one-way ANOVA with Bonferroni’s post-hoc test: *p < 0.05, **p < 0.01, ***p < 0.001, ****p < 0.0001 compared to the untreated cells (control).

The termination of cell proliferation is often associated with the triggering of several cell death pathways, therefore, experiments measuring the fluorescence of apoptotic cells were performed. These results are consistent for both cell lines and show a clear increase in both early and late apoptosis at approximately 60% after incubation with IS20 (Fig. 8). These results indicate a strong activation of apoptotic processes by IS20.

**Fig. 9:**
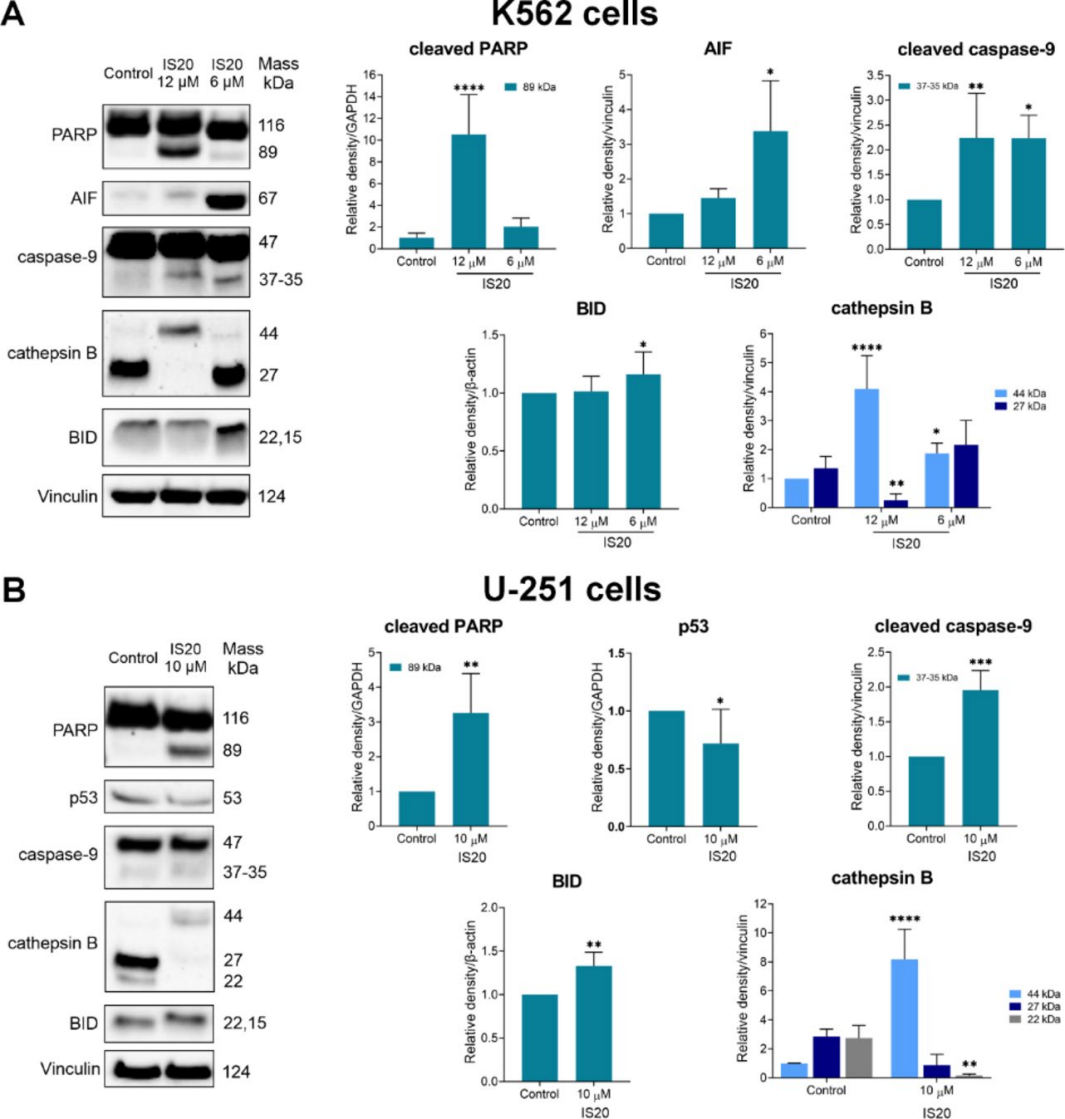
The impact of IS20 on the induction of expression of apoptosis-related proteins in the K562 (**A**) and U-251 (**B**) cells. Results were normalized to the reference protein and are from five independent experiments (*n* = 5). Immunoblots shown are representative from these independent experiments. The displayed blots were cropped, and the full-length blots are shown in Fig. S6. The statistical analysis of the data was performed using a one-way ANOVA with Bonferroni’s post-hoc test: *p < 0.05, **p < 0.01, ***p < 0.001, ****p < 0.0001 compared to the untreated cells (control).

The next step was to analyze apoptotic-related proteins (Fig. 9). One of the indicators of the apoptosis process is the presence of cleaved PARP and caspase-9 proteins. Densitometric analysis indicated a 10-fold increase in the concentration of the PARP cleavage fragment with a molecular weight of 89 kDa following administration with 12 µM of IS20 incubated with the K562 cell line. The U-251 cell line showed a more than 3-fold increase in the concentration of this cleavage product. Moreover, IS20 also influenced the activation of the cell death pathway through caspase-9 cleavage. In both cell lines, an approximately 2-fold increase in the 37 − 35 kDa molecular weight caspase-9 cleavage product was observed compared to control cells. Apoptosis-inducing factor (AIF) protein is involved in triggering a caspase-independent apoptotic pathway^76^. Therefore, we decided to test if the mechanism of IS20 action also occurs through a pathway independent of caspase involvement. Western Blot results indicate that this protein was activated more than 3-fold in the leukemia line for an IS20 concentration of 6 µM. This indicated a multifaceted action of the tested derivative. Interestingly, the AIF protein was undetected in the glioblastoma line. While for the K562 cell line, p53 was undetected due to the loss of one allele and insert mutation in the second allele^30,77^, the U-251 cell line gave an unusual response. Many papers on 2-styrylquinazolines, including CP-31398, showed overexpression of p53 protein^11^, while our study shows a decrease in activity. This is noteworthy because of the elevated p21 level in both lines and indicates an important role for the p53 protein in the cell’s response to treatment. Cathepsin B is a lysosomal cysteine peptidase involved in programmed cell death. This protein is initially synthesized as an inactive proenzyme form (44 kDa), and modified into an active two-chain form (27 and 24 kDa)^78^. The intrinsic apoptotic pathway activated by increased ROS levels initiates leakage of cathepsin B from the lysosome, resulting in BID activation^79^. A marked (8-fold for U-251 and 4-fold for K562) increase of procathepsin B (44 kDa) was observed for both cell lines incubated with IS20. Additionally, the heavy chain form of cathepsin B demonstrated a marked decrease. BID belongs to a pro-apoptotic member of the Bcl-2 protein family, and its expression is upregulated by the tumor suppressor p53^80^. Upregulation of BID was observed in both leukemia and glioblastoma cell lines, which confirms the pro-apoptotic mechanism of IS20.

### ADME properties

In the early stage of the drug discovery process, especially orally administered drugs, it is important to perform at least indicative absorption, distribution, metabolism, and excretion (ADME) profiling to provide critical information about the basic physiologic behavior of a potential drug. ADME screening is generally performed using software-based approaches, and ADME profiling is subsequently verified for selected drug candidates in preclinical and clinical studies^81,82^.

The Lipinski Rule of Five (Ro5) is one of the most accepted recommendations concerning the physicochemical parameters of biologically active compounds, and all medicinal chemists try to follow it when designing molecules^83^. Ro5 contains the limits of specific molecular descriptors (MW < 500, logP < 5, HBD < 5, HBA < 10) set based on experimentally and statistically obtained results so that a compound that meets this recommendation has a higher chance of becoming a drug. However, a good drug-like score does not make a molecule a drug, and vice versa^84,85^. It is clear that ADME-friendly properties, such as lipophilicity, polar surface area, etc., are important in the context of specific ligand-receptor interactions.

The following table (Table 1) shows the predicted ADME-influencing properties of IS20 compared to those of the model tyrosine kinase inhibitors (TKIs) presented in the paper, i.e., gefitinib, erlotinib, lapatinib, afatinib, KIN-281, KIN-236 and CP-31398 with an *N*-phenylquinazolin-4-amine scaffold. Also, imatinib was chosen as the comparator drug due to its ability to inhibit ABL, although the *N*-[4-(pyridin-3-yl)pyrimidin-2-yl]benzene-1,3-diamine fragment is in its molecule. TKIs are known to have limited bioavailability, tend to be extensively metabolized, and are substrates of P-glycoprotein^86^, therefore, in addition to Ro5 parameters, intestinal absorption and permeation into the brain were also characterized.

**Table 1:** Values of parameters characterizing physicochemical properties of discussed TKIs predicted using ACD/Percepta ver. 2012.

Comp.	MW	logP	HBD	HBA	RB	TPSA [Å^2^]	Parachor [cm^3^]	k_a_ [min^− 1^]	logBB	logPS	Brain plasma equilibrim rate
Imatinib	493.60	3.10	2	8	7	86.28	1110.03	0.043	-0.16	-2.0	-3.1
Gefitinib	446.90	3.70	1	7	8	68.74	921.04	0.049	0.12	-1.4	-2.9
Erlotinib	393.44	3.05	1	7	11	74.73	877.68	0.050	-0.84	-1.5	-2.6
Lapatinib	581.06	5.22	2	8	11	114.73	1159.20	0.046	-0.50	-1.4	-3.5
Afatinib	485.94	3.44	2	8	8	88.61	980.17	0.044	-0.46	-2.0	-3.3
KIN-281	492.87	6.01	3	5	9	67.07	1027.98	0.047	0.31	-2.3	-4.4
KIN-236	503.46	5.99	1	6	11	59.51	1079.62	0.047	0.41	-2.0	-4.2
CP-31398	362.47	3.72	1	5	8	50.28	834.47	0.048	0.00	-1.7	-3.2
IS20	397.43	4.59	1	6	5	65.50	839.46	0.051	-0.33	-1.1	-3.1

Molecular weight (MW), lipophilicity (log P), number of H-bond donors (HBD), number of H-bond acceptors (HBA), number of rotatable bonds (RB), topological polar surface area (TPSA), absorption rate in human jejunum at pH 6.5 (*k*_*a*_), permeability across BBB (logBB - concentration of drug in brain divided by concentration in blood), rate of brain penetration (logPS - permeability surface-area product).

IS20 has a molecular weight (MW) close to erlotinib (MW approx. 400). Lipinski’s recommendation of MW < 500 is not met by lapatinib and KIN-236. Limit values also have imatinib, KIN-281 and afatinib. The compounds KIN-281, KIN-236, and lapatinib do not meet the recommendation of Ro5 for lipophilicity (logP < 5). IS20 has a logP value of ca. 4.6, so it is the fourth most lipophilic molecule from Table 1. All the compounds meet the criteria for the number of H-bond donors (HBD) and acceptors (HBA), and in this regard, IS20 is close to KIN-236, gefitinib and erlotinib. The number of rotatable bonds (RB) of IS20 is close to imatinib, while the topological polar surface area (TPSA) of IS20 is close to KIN-281 and gefitinib, and parachor is close to its parent CP-31398. TPSA has been recognized as a good indicator of intestinal drug absorption, with the acceptable limit for good absorption being a TPSA value < 120 Å^2^^87^. In addition, for orally administered compounds, which are intended to cross the blood-brain barrier (BBB), the TPSA value should be lower than 70 Å^2^^88^. The TPSA value of IS20 is 65.50 Å^2^, which suggests that the compound should have good intestinal absorption. The predicted value of the rate of absorption in the jejunum (*k*_*a*_ = 0.051 min^− 1^) of IS20 is closest to erlotinib and is the highest of all the evaluated compounds.

A remarkable prediction was made by ACD/Percepta for BBB permeation. In general, logBB ≥ 0.3 is for BBB permeable drugs and logBB ≤-0.3 is for impermeable drugs^89^, the value of logBB = -0.33 for IS20 is borderline and lies between the predicted values for imatinib (logBB = -0.16) and lapatinib (logBB = -0.50). More informative are the values of the permeability surface-area product (expressed as logPS)^90^, which for IS20 is logPS = -1.1 and thus close to lapatinib and gefitinib (logPS = -1.4). For all three drugs (imatinib, lapatinib, and gefitinib), the probability of penetration through the BBB into the brain ranges from 0.62 to 0.97 (values are taken from the DrugBank database). IS20 has some parameters similar to erlotinib, whose BBB permeation probability is 0.93 (value from DrugBank), and the brain plasma equilibrium rate predicted by ACD/Percepta for IS20 is -3.1 and is equal to the rate predicted for imatinib. However, the ABL inhibitor is a strong substrate for efflux via P-glycoprotein and has limited effect on the brain. Conversely, the value for IS20 is quite close to afatinib (-3.3), which can penetrate the BBB in preclinical models and is sufficient for action in the central nervous system (CNS). Therefore, IS20 may also be predicted to be able to reach therapeutic levels in the CNS. However, more detailed studies on relevant cellular models^91^ are needed.

### *In vivo s*tudies

To elucidate the toxicological reactions of organisms to IS20, the *Danio rerio* experimental model was employed as a reliable and rapid *in vivo* tool for toxicity assessment. Zebrafish possess several advantageous characteristics, including economical housing requirements, diminutive size reducing space and test agent consumption, and a high fecundity rate, with individual females capable of generating approximately 300 eggs, thereby supporting their efficiency as a model organism. Moreover, zebrafish and humans share an approximate 70% genomic similarity. Notably, there exists considerable conservation of critical developmental and physiological processes, such as those governing the digestive, nervous, and cardiovascular systems, when compared to humans. This conservation largely underpins the robust parallels in response to pharmacological agents between the two species, with numerous zebrafish models accurately recapitulating human diseases both genetically and phenotypically^92^. Indeed, zebrafish have attained widespread recognition as a primary model organism within the domain of high-throughput screening for biological investigations, akin to conventional models such as mice and rats^93^.

**Fig. 10:**
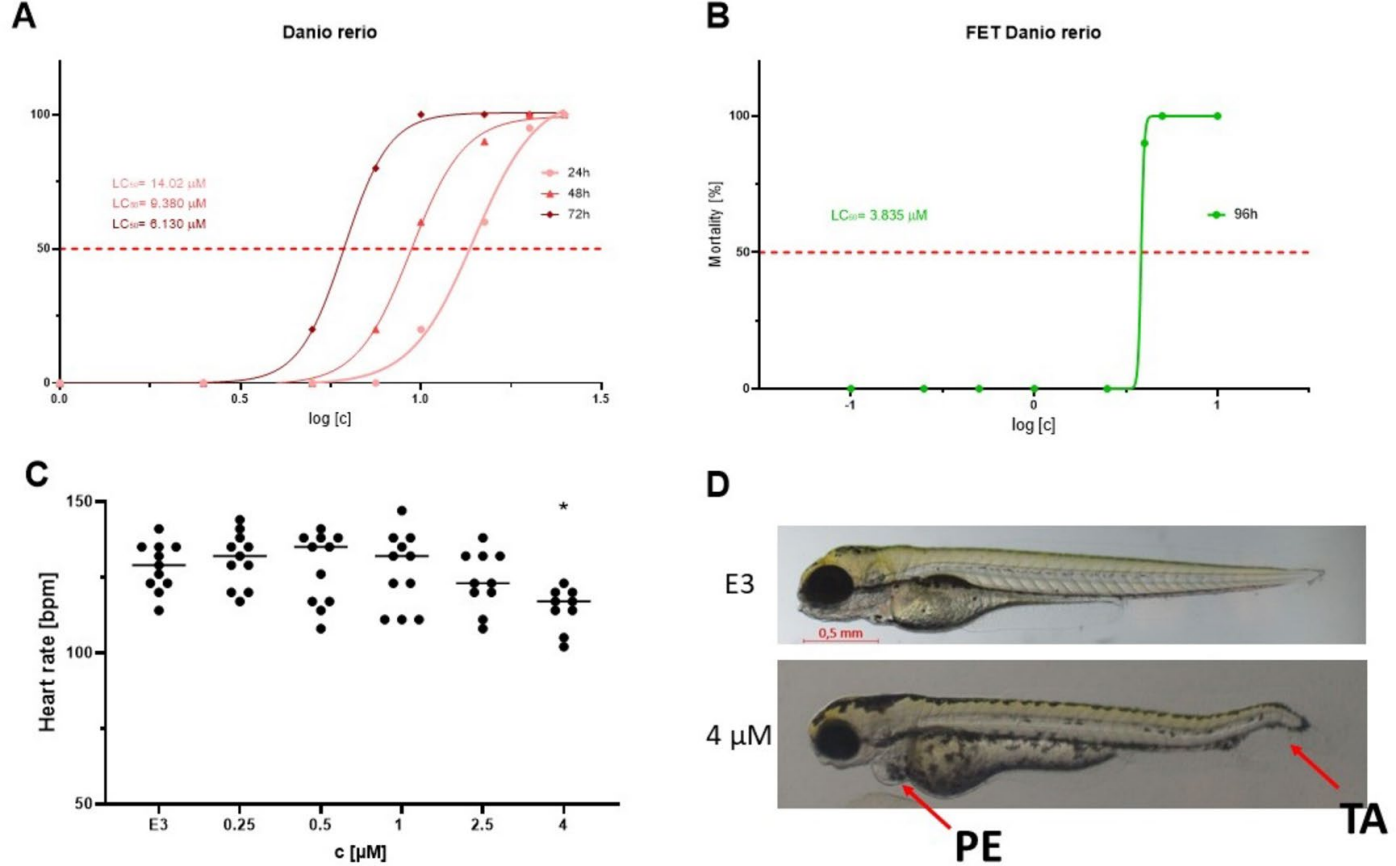
The toxicity of IS20 in *Danio rerio* experimental model. (**A**) Toxicity (expressed as LC_50_) after 24, 48 and 72 h of incubation with IS20; (**B**) Toxicity (expressed as LC_50_) of IS20 in FET after 96 h of incubation; (**C**) Cardiotoxicity, measured as heart rate [beats per minute], the statistical analysis of the data was performed using a one-way ANOVA with Tukey’s post-hoc test: *p < 0.05 compared to the untreated control (E3) fish; (**D**) Example of malformations stated for the incubation with IS20 versus untreated control (E3) fish; *PE* pericardial edema, *TA* tail autophagy.

The toxicological evaluation of IS20 was performed according to the OECD 236 test. The freshly fertilized zebrafish eggs were exposed to different concentrations of IS20. Every 24 h, the embryos were observed for visual indicators of lethality like the coagulation of fertilized eggs, lack of somite formation, lack of detachment of the tailbud from the yolk sac and lack of heartbeat. The obtained outcomes showed that the toxicity of IS20 increased with time of incubation from 14.02 µM (24 h: Fig. 10A) to 3.85 µM (96 h: Fig. 10B).

Zebrafish present an attractive platform for cardiovascular risk assessment post-treatment due to the straightforward nature of heart rate measurement. Notably, zebrafish exhibit remarkable resilience, as they can persist without cardiac output and despite major vascular anomalies for extended periods, a feat not commonly observed in larger animal models. These attributes have collectively propelled *Danio rerio* into the spotlight for evaluating cardiotoxicity and cardiovascular developmental effects following drug administration^94^.

As an indicator of normal heart functions, heartbeats were measured after incubation of 96 h. Interestingly, a small decrease in heart rate was observed only at the highest dose (Fig. 10C). Additionally, at that dose of IS20, we observed developmental malformations, which are a consequence of some heart dysfunctions (Fig. 10D). Pericardial edema (PE) is commonly observed in zebrafish embryo-based chemical toxicity screens, with the underlying mechanism potentially related to disruption of embryonic osmoregulation. Tail autophagy (TA) is closely related to the disruption of circulation related to deregulation of heart functions.

## Conclusions

Unlike our previous investigations in which we presented large libraries of compounds searching for a privileged structure, in this paper, we present IS20, which is attractive in terms of its multi-target mechanism of action. The selected cell model included cell lines with different p53 status, which allowed us to analyze the effect of this protein on different molecular targets. This work aimed to conduct a detailed analysis of the mechanism of action of the antiproliferative activity of the IS20 derivative based on previous results showing interesting inhibitory potential for tyrosine kinases, particularly ABL and SRC. Notably, this is the first report to indicate that a 2-styrylquinazoline compound can bind to iron and exhibit chelating properties, thereby affecting the generation of ROS. Disruption of cellular homeostasis by IS20 triggered several factors, including antioxidants such as GSH, *MnSOD* and *CAT*, associated with cellular defense against the induction of oxidative stress. This, in turn, affected the activation of HO-1 and HIF-1α proteins. Interestingly, the *Ndrg1* gene was overexpressed and inhibited the EGFR/mTOR signaling pathway. The final effect of IS20 was the activation of coexisting cell death pathways apoptosis and autophagy, which was confirmed by the activation of the cascade of proteins responsible for these processes. Based on the prediction from the primary ADME screening using the commercially available ACD/Percepta software, it could be assumed that the investigated compound may have suitable physicochemical parameters for sufficient bioavailability. The *in vivo* studies on the *Danio rerio* model evaluated the toxicological responses of IS20 on living organisms. The lethal doses were decreased with incubation time, receiving an LC_50_ value of approximately 3.85 µM at the end of the exposure period, i.e., at 96 hpf. The compound was not found to be cardiotoxic up to the LD_50_ dose. Moreover, no lethal malformations were observed, only those connected with lower heart rates at the highest survived dose (4 µM), namely PE and TA. Further chemical modifications should be considered to fully avoid or at least diminish the cardiotoxicity. Based on the structure of the IS20 as well as its profile of activity, ROS generation and excessive oxidation stress can be regarded as factors connected with cardiotoxicity. According to our former study the chelation of iron may be a key feature for oxidative toxicity. This is connected with higher lipophilicity, which can strengthen the toxicity against circulatory system and also with ionophoric activity^37,38^. From^13^, some less lipophilic styrylquinazolines are known to still possess good multitarget anti-kinase activity. Particularly, exchange the benzodioxole to methoxylated phenyl group would be beneficial. On the other hand, in^19^, we presented less lipophilic sulfonates of C4 quinazoline core with good activity, which may suggest a possible route. Further modifications that will be considered are the bioisosteric exchange of the 4-amine-quinazoline group that may be involved in the chelation of divalent metal ions and may therefore participate in oxidizing stress.

## Materials and methods

### Chemicals

The metal salts, such as iron(III) chloride hexahydrate and copper(II) sulfate pentahydrate, were purchased from POCH and Chempur, respectively. DMSO (99.9% purity) for spectroscopy studies was obtained from Thermo Scientific. Imatinib was acquired from Sigma-Aldrich. The IS20 compound (*N*-(1,3-benzodioxol-5-yl)-2-[(*E*)-2-(2-methoxyphenyl)vinyl] quinazolin-4-amine) was synthesized and characterized as described previously^13^.

### Cell culture

The human glioblastoma cell lines T98G, LN-18, LN-229, and U-87MG were purchased from ATCC. The glioblastoma cell line U-251 was kindly provided by Prof. G. Kramer-Marek from the Institute of Cancer Research in London, United Kingdom. The human suspension chronic myelogenous leukemia cell line K562 was purchased from Sigma-Aldrich. All adherent cancer cell lines were cultured in Dulbecco’s modified Eagle’s medium (DMEM) that had been supplemented with 10% heat-inactivated fetal bovine serum (FBS) (all from Sigma-Aldrich) in 75 cm^2^ flasks (Nunc). The suspension cell line K562 was cultured in an RPMI-1640 medium (Sigma-Aldrich), which contained 10% heat-inactivated FBS. Each medium contained a combination of penicillin and streptomycin (1% v/v; Gibco). All cell lines were cultured under standard conditions at 37 °C with a 5% CO_2_ humidified atmosphere. Moreover, all cell lines were routinely tested for mycoplasma using the PCR technique with specific *Mycoplasma* primers to confirm that there was no contamination.

### Cell treatments

The IS20 compound was dissolved in DMSO at a concentration of 8.35 mM, then diluted in medium (DMEM or RPMI-1640) to a concentration of 25 µM (final DMSO concentration did not exceed 0.3%). For cytotoxicity assays, cells were incubated with IS20 in various concentrations (0–25 µM). For the remaining assays, K562 cells were incubated with IS20 at 12 µM and 6 µM, and the U-251 cells were exposed to IS20 at 10 µM. The doses of IS20 for these experiments were selected based on the results of antiproliferative activity and calculated IC_50_ values. Imatinib was used for autophagy assays, which was dissolved in DMSO at a concentration of 8.35 mM, then diluted in DMEM or RPMI-1640 medium to concentrations of 25 µM or 0.4 µM, respectively.

### Cytotoxicity studies

The cells were seeded in 96-well plates (Nunc) at a density of 5,000 cells per well and incubated under standard conditions at 37 °C for 24 h. The assay was performed following a 72 h incubation with IS20, according to standard methodology as described previously^13,19^. Briefly, 100 µL of DMEM without phenol red containing 20 µL of the CellTiter 96^®^AQueous One Solution-MTS (Promega) solution was added to each well and incubated for 40 min–1 h at 37 °C. The optical densities of the samples were measured at 490 nm using a multi-well plate reader (Varioskan LUX, Thermo Scientific). The results were compared to the control and were estimated as the inhibitory concentration (IC_50_) values (using GraphPad Prism 9). Each compound was tested in triplicate in a single experiment, with each experiment being performed at least four times.

### Intracellular measurement of ROS levels

The U-251 and K562 cells were seeded in 96-well plates at a density of 9,000 per well and incubated with the freshly prepared solutions of IS20 for 3, 6, 9, 12 and 24 h. Additionally, the cells were incubated for 24 h with a solution of IS20 with ascorbic acid (100 µM). The ROS levels were measured immediately using the CellROX^®^ Green Reagent assay procedure. In addition, Hoechst 33,342 (Molecular Probes™) staining was used to determine the number of cells in each well. Briefly, after treatment, the medium was removed (plates with K562 cells had been previously centrifuged), and a 100 µL mixture of CellROX Green Reagent (5 µM) and Hoechst 33,342 (6 µM) was added to each well. The samples were incubated for 30 min under standard conditions in the dark. The fluorescence was measured using a multi-plate reader (Synergy 4, Bio Tek), and readings were performed at 485 nm excitation and 520 nm emission, and 345 nm excitation and a 485 nm emission for CellROX Green Reagent and Hoechst 33,342, respectively. ROS levels were calculated as the percentage relative to the untreated controls. The experiments were performed at five times.

### Time-dependent measurement of GSH level

The U-251 and K562 cells were seeded in 3 cm Petri dishes (Nunc) at a density of 500,000 cells per well. The next day, the medium was replaced by freshly prepared solutions of IS20. The cells were incubated with this derivate at the same times as the levels of intracellular ROS measurement. Preparation of the lysates and intracellular glutathione levels were determined by the method described by Rahman et al.^95^. In short, the lysates in a volume of 20 µL were transferred into 96-well plates. Then, freshly prepared DNTB (0.67 mg/mL) and glutathione reductase (1.67 units/mL) solutions in KPE buffer were added to each well and incubated for 30 s. Immediately afterward, a solution of β-NADPH (0.67 mg/mL) prepared in KPE buffer was added to each well. The samples were mixed, and the absorbance was measured immediately at 412 nm in a Synergy4 multi-well plate reader. Data were compared to the levels of GSH in untreated cells (control). The experiments were repeated at five times.

### Cell cycle assay

The K562 and U-251 cells wereseeded in 3 cm Petri dishes at a density of 250,000 cells per well and incubated under standard conditions. After a day, the medium was removed and replaced by freshly prepared solutions of IS20. After a 24-hour incubation, the assays were performed using a Muse Cell-Cycle Kit (Millipore) according to the manufacturer’s instructions. In short, cells were collected, washed with cold PBS and centrifuged at 300 g for 5 min. Next, the cells were fixed in ice-cold 70% ethanol and stored at -20 °C overnight. The following day, the cells were washed with cold PBS and centrifuged. Then, the cell pellets were resuspended in 200 µL of Muse™ Cell Cycle Reagent. Samples were incubated for 30 min at room temperature in the dark. The analysis of cellular subpopulation values in individual cell cycle phases was estimated using a Muse Cell Analyzer (Millipore). The experiments were performed at least four times.

### Apoptosis assay

The K562 and U-251 cells were seeded in the same manner as for the cell cycle assay. After a day, the medium was removed and replaced by freshly prepared solutions of the tested compound IS20. The samples were incubated for 48 h. Next, the assays were performed using the FITC-Annexin V Apoptosis Detection KIT with 7-AAD (Bio-Legend) according to the manufacturer’s instructions. In short, the cells were collected, washed twice with cold PBS and centrifuged at 300 g for 5 min. Then, the pellets of cells were resuspended in 100 µL Annexin V Binding Buffer. Next step, 5 µL of FITC-Annexin V and 5 µL 7-AAD Viability Staining Solution were added to each sample and incubated for 15 min at room temperature in the dark. After immunostaining, the number of events for live, early and late apoptotic cells was determined using a Muse Cell Analyzer. The experiments were performed at least four times.

### Autophagy assay

The K562 and U-251 cells were seeded in 96-well plates and incubated at 37 °C for 24 h. The cell density was 20,000 cells per well (for the 24 h assay) and 10,000 per well (for the 48 h assay). Next, the medium was changed for freshly prepared solutions of IS20 and the cells were incubated for 24–48 h. Additionally, the cells were treated with imatinib as the positive control. After treatment, the assays were performed using a Muse™ Autophagy LC3-antibody-based kit (Millipore) according to the manufacturer’s instructions. Briefly, the cells were collected, washed with cold Hank’s Balanced Salt Solution and centrifuged at 300 g for 5 min. Then, the cells were resuspended in a mixture of 95 µL 1X Autophagy Reagent B and 5 µL Anti-LC3 Alexa Fluor^®^555 antibody. The samples were incubated on ice for 30 min in the dark. After incubation, the cells were centrifuged and resuspended in 200 µL of a 1X Assay Buffer. Then, the samples were directly processed to analyze the autophagy induction using a Muse Cell Analyzer. The autophagy induction ratio was calculated based on the ratio between the target’s fluorescence versus the control sample. The experiments were performed at five times.

### qRT-PCR studies

The total RNA was isolated from the K562 and U-251 cells after 24-h treatment with IS20 according to the TRIzol Reagent procedure (Ambion). Reverse transcription was performed with 5 µg of total RNA using a GoScript™ Reverse Transcriptase kit (Promega) and Oligo(dT)_23_ Primers (Sigma). The Real-Time PCR was carried out in a 10 µL reaction volume using CTX96 Touch™ Real-Time PCR Detection System (Biorad). Each sample contained SsoAdvanced™ Universal SYBR^®^ Green Supermix (Biorad), a specific primer pair mix (0.5 µM each) and 1 µL of cDNA. The reaction was performed under the following conditions: initial denaturation at 95 °C for 30 s; followed by 40 denaturation cycles at 95 °C for 15 s; annealing (primer-specific temperature for 30 s) and extension at 72 °C for 60 s. The results were analyzed based on a comparison of the expression of the target genes to a reference gene (*GAPDH)* using the 2^−ΔΔCT^ method. The experiments were performed at least four times.

### Immunoblotting studies

The K562 and U-251 were seeded in 3 cm Petri dishes at a density of 500,000 cells per well and incubated under standard conditions for 24 h. Next, the cells were incubated with freshly prepared solutions of IS20 for one day. After this time, the U-251 cells were detached by trypsinization. In turn, K562 were immediately collected into Eppendorf tubes. All samples were centrifuged at 2,000 rpm. Next, the cell pellets were resuspended in a RIPA buffer containing Halt Protease Inhibitor Cocktail, Halt Phosphatase Inhibitor Cocktail along with 0.5 M EDTA (all from Thermo Scientific) and lysed on ice for 20 min. The obtained lysates were sonicated and centrifuged at 10,000 rpm for 10 min at 4 °C. The supernatants were transferred to new tubes and used in further studies. The protein concentration was measured using a Micro BCA™ Protein Assay Kit (Thermo Scientific) according to the manufacturer’s instructions. 16 µg of the proteins were electrophoresed on SDS-Page gels and transferred onto nitrocellulose membranes. The membranes were blocked in 5% non-fat milk prepared in TBST (containing 0.1% Tween-20) for 1 h. After this time, the membranes were incubated with specific primary antibodies (all from Cell Signalling Technology) at 1:1000 dilution for target proteins: cyclin E1 (#4129), cdc2 (#9116), p21^Waf1/Cip1^ (#2947), PARP (#9542), AIF (#5318), caspase-9 (#9508), cathepsin B (#31718), BID (#2002), p53 (#2524), HO-1 (#5853), HIF-1α (#14179), EGFR (#4267), phospho-EGFR (Tyr1068) (#2236), mTOR (#2983), phospho-mTOR (Ser2448) (#5536), phospho-Akt (Ser473) (#4060), phospho-p44/42 MAPK (Erk1/2) (Thr202/Tyr204) (#9101), as well as at 1:2000 dilution for reference proteins: GAPDH (#2118), β-actin (#3700) and vinculin (#13901) overnight at 4° C. The following day, membranes were washed in TBST and incubated with horseradish peroxidase (HRP)-conjugated secondary antibodies for 1 h at room temperature. Then, the membranes were washed in TBST and incubated with a SuperSignal™ West Pico Chemiluminescent Substrate (Thermo Scientific). The chemiluminescence signals were captured using a ChemiDoc™ XRS + System (BioRad). The experiments were repeated at least five times. The densitometric analysis was performed using ImageJ software (Wayne Rasband, National Institutes of Health, USA).

### Lumit immunoassay

The U-251 and K562 cells were seeded into 96-well plates at a density of 50,000 for 24 h before the treatment. Next, the medium was changed for freshly prepared solutions of IS20 and the cells were incubated for 24 h. At the same time, Fluorogenic Live Cell Substrate (GF-AFC Substrate) was added for the final concentration in each well of 50 µM. Lumit immunoassay was done following the manufacturer’s instructions. In brief, lysis buffer containing digitonin was mixed with the immunoassay reaction buffer and added to the wells. The plates were then mixed for 20 min. After that, primary antibodies against p-EGFR (Tyr1068), mTOR, p-mTOR (Ser2448) and Lumit secondary antibodies were added to the wells. The plates were then incubated at 23 °C for 90 min, followed by adding furimazine substrate. Luminescence and fluorescence were measured after 2 min using a multi-plate reader Varioskan LUX. The instrument was set to 1 s time. Tests were done at least three times with duplicate repeats on each plate.

### *In vivo* toxicity

The fish embryo toxicity (FET) test was performed on zebrafish - *Danio rerio* (Experimental Medicine Centre, Medical University of Lublin, Poland) according to OECD Test Guideline 236 to determine the toxicity of IS20. According to the procedure, newly fertilized zebrafish eggs were exposed to the solution of IS20 for a period of 96 h at concentrations ranging from 1 to 25 µM. The system applied in the experiment was static, as the changes in solution concentrations did not exceed the range of 20% of nominal concentration values. The solutions were prepared in normal embryo culture medium - E3 solution (5 mmol/L NaCl, 0.17 mmol/L KCl, 0.33 mmol/L CaCl_2_, and 0.33 mmol/L MgSO_4_, containing no methylene blue, with a pH value of about 7.2). The experiment was performed in 24-well plates, with five embryos per well, and ten per group. Each plate was covered and kept in an incubator set at 28 ± 0.5 °C under a light/dark period of 12/12 h. Every 24 h, acute toxicity was determined based on a positive outcome in any of the four visual indicators of lethality, including the coagulation of fertilized eggs, lack of somite formation, lack of detachment of the tailbud from the yolk sac and lack of heartbeat. The values of LC_50_ for each timepoint (24, 48, 72 and 96 h) were calculated using GraphPad Prism 10. The final value of toxicity is the one measured at the end of the exposure period (96 hpf - hours post fertilization). Cardiotoxicity, as defined by heart rate, was also evaluated at that time point.

Experiments using the *Danio rerio* model were carried out on larvae up to 96 h post-fertilization, and therefore the approval of Ethics Committees was not required (according to Polish law and EU directives). All experiments using the *in vivo* model were carried out respecting the 3Rs principle and other guidelines (e.g. ARRIVE) for working with animals.

### Tyrosine kinase assay

To determine the inhibition ofnon-receptor tyrosine kinases, assays were performed using the kinase selectivity system TK-1 and the ADP-Glo Kinase Assay (both from Promega). The protocol was developed by our group and previously described^13,32^. Experiments were performed at least four times. Data are expressed as a percentage of tyrosine kinase inhibitory activity after treatment with the derivatives tested.

### Studies of the complexing properties of IS20 with metal ions

The tested compound and metal ions (Fe (III) and Cu (II)) were dissolved in DMSO to obtain an initial stock solution of 100 µM. Then, solutions of the tested IS20 derivative and ions were prepared by transferring the appropriate volume into individual wells of a 96-well black plate with a transparent bottom (Nunc). Finally, each well contained 200 µL of solution containing: the IS20 derivative at a concentration of 50 µM and various concentrations of ions to obtain the following ligand-to-metal molar ratios: 1:0; 10:1; 5:1; 3.3:1; 2.5:1; 2:1; 1.67:1; 1.43:1; 1.25:1; 1.11:1; 1:1. The prepared plates were then incubated for 4 h at room temperature. Absorption spectra in the range of 275 to 450 nm (in 5 nm steps) were measured using a multi-well plate reader Varioskan LUX. All spectra were normalized using vector normalization to reveal the hidden isosbestic point (HIP). Absorption spectra were processed by OriginPro 2023 software.

### ADME calculations

All parameters were predicted using the commercially available program ACD/Percepta ver. 2012 (Advanced Chemistry Development, Inc., Toronto, ON, Canada, 2012).

### Statistical analysis

The obtained results are presented as the mean ± standard deviation (SD) from 3 to 6 independent experiments. Depending on the type of experiment, the statistical analysis was carried out using the one-way ANOVA with a Bonferroni post-hoc test or unpaired t-test. Lumit Immunoassay statistical analysis was done with the usage of one-way ANOVA with a Dunn–Šidák correction test. A p-value of 0.05 or less was considered statistically significant. All statistical tests were performed using GraphPad Prism 9 software (GraphPad Software, USA).

## Electronic supplementary material

Below is the link to the electronic supplementary material.

Supplementary Material 1

## Data Availability

Data supporting the findings of this study are available within the article. Source data are available from the corresponding author on request.
