# Supplementary material for: Unveiling the role of Ndrg1 gene on the oxidative stress induction behind the anticancer potential of styrylquinazoline derivatives
Source: Sci Rep. 2025 May 8;15:16081. doi: 10.1038/s41598-025-99277-1 (PMC12062220; doi:10.1038/s41598-025-99277-1)
Supplement: Supplementary file 1 — Supplementary Material 1 [file 41598_2025_99277_MOESM1_ESM.docx]

**Unveiling the role of *Ndrg1* gene on the oxidative stress induction behind the anticancer potential of styrylquinazoline derivatives**

**Katarzyna Malarz^1,2*^, Michał Kuczak^3^, Patryk Rurka^2^, Patrycja Rawicka^2^, Anna Boguszewska-Czubara^4^, Josef Jampilek^5^, Jacek Mularski^3^, Robert Musiol^3^, Anna Mrozek-Wilczkiewicz^1,2^**

^1^Department of Systems Biology and Engineering, Silesian University of Technology, Akademicka 16, 44-100 Gliwice, Poland

^2^Institute of Physics, University of Silesia in Katowice, 75 Pułku Piechoty 1a, 41-500 Chorzów, Poland

^3^Institute of Chemistry, University of Silesia in Katowice, 75 Pułku Piechoty 1a, 41-500 Chorzów, Poland

^4^Department of Medical Chemistry, Medical University of Lublin, Chodźki 4a, 20-093 Lublin, Poland

^5^Department of Chemical Biology, Palacky University Olomouc, Slechtitelu 27, 779 00 Olomouc, Czech Republic

*corresponding author: katarzyna.malarz@polsl.pl, katarzyna.malarz@us.edu.pl

**Table of contents:**

[**Table S1.** Characterization of the p53 protein in the GBM cell lines as well as K562 cells. 2](#_Toc193473201)

[**Table S2.** Inhibitory enzymatic activity of the IS20 2](#_Toc193473202)

[**Table S3.** The percentage of live, early and late apoptotic cells 2](#_Toc193473203)

[**Table S4.** Sequences of primer pairs used in determining the mRNA expression 3](#_Toc193473204)

[**Figure S1.** Effect of IS20 (IC_50_ concentration) and a combination IS20 with Vitamin C (100 uM) on reactive oxygen species (ROS) generation after 24 h (A) and cellular proliferation after 72 h (B) in U-251 and K562 cells. 3](#_Toc193473205)

[**Figure S2.** Spectroscopic titrations spectra of IS20 with Fe^3+^ (A) and Cu^2+^ (B) ions 4](#_Toc193473206)

[**Figure S3.** The expression levels of p-EGFR, mTOR and p-mTOR after exposure to IS20 4](#_Toc193473207)

[**Figure S4.** The influence of IS20 on the cell cycle arrest 5](#_Toc193473208)

[**Figure S5.** The influence of IS20 on the induction of autophagy 6](#_Toc193473209)

[**Figure S6.** The uncropped Western blots 14](#_Toc193473210)

**Table S1.** Characterization of the p53 protein in the GBM cell lines as well as K562 cells.

| Cell line | p53 | | |
| --- | --- | --- | --- |
|  | status | position | effect |
| **U-251** | missense mutation | codon 273 | substitution of arginine for histidine |
| **T98G** | missense mutation | codon 237 | substitution of methionine for isoleucine |
| **LN-18** | missense mutation | codon 238 | substitution of cysteine for serine |
| **LN-229** | missense mutation | codon 98 | substitution of proline for leucine |
| **U87-MG** | wild-type | - | - |
| **K562** | loss of one allele and insert mutation in second allele | exon 5 | truncated form with 148 amino acids |

**Table S2.** Inhibitory enzymatic activity of the IS20 ­tested against receptor tyrosine kinases.

| **compound** | **Inhibition of tyrosine kinase activity [%]^*^** | | | | |
| --- | --- | --- | --- | --- | --- |
|  | **EGFR** | **HER2** | **HER4** | **IGF1R** | **InsR** |
| IS20 | 24.84 ± 5.43 | 30.42 ± 9.89 | 20.40 ± 2.77 | 39.20 ± 9.62 | 35.81 ± 10.09 |

* measurement for IS20 at a concentration of 1 µM

**Table S3.** The percentage of live, early and late apoptotic cells after incubation with IS20 in K562 and U-251 cells from all performed experiments. Data are presented as mean ± SD.

|  | **K562** | | **U-251** | |
| --- | --- | --- | --- | --- |
|  | **Control** | **IS20**  **12 µM** | **Control** | **IS20**  **10 µM** |
| **Live [%]** | 92.98 ± 2.26 | 37.70 ± 6.48 | 81.72 ± 7.64 | 40.03 ± 10.59 |
| **Early apoptotic [%]** | 4.92 ± 2.11 | 18.31 ± 6.77 | 5.15 ± 3.81 | 32.30 ± 1.07 |
| **Late apoptotic [%]** | 1.10 ± 0.84 | 40.53 ± 15.91 | 5.70 ± 2.98 | 27.71 ± 3.95 |

**Table S4.** Sequences of primer pairs used in determining the mRNA expression of tested genes.

| Gene | Forward primer (5’→3’) | Reverse primer (3’→5’) | |
| --- | --- | --- | --- |
| *MnSOD* | ACAAACCTCAGCCCTAACGG | | TGCTCCCACACATCAATCCC |
| *CAT* | ACTGTTGCTGGAGAATCGGG | | AAGTCTCGCCGCATCTTCAA |
| *IDH-1* | TCCGTCACTTGGTGTGTAGG | | GGCTTGTGAGTGGATGGGTA |
| *Ndrg1* | GTCTCGGGAGATGCAGGATG | | TGTGGTTCATGCCGATGTCA |
| *Calreticulin* | CTGCCGTCTACTTCAAGGA | | GAACTTGCCGGAACTGAGAAC |
| *GADD45* | AGTCAGCGCACGATCACTGT | | GGATCAGGGTGAAGTGGATCT |
| *LC3* | CAGCATCCAACCAAAATCCC | | CACTGACAATTTCATCCCGAAC |
| *p62* | AGGACGGGGACTTGGTTG | | GGCGGGAGATGTGGGTAC |
| *GAPDH* | GAGTCAACGGATTTGGTCGTA | | GCCCCACTTGATTTTGGAG |


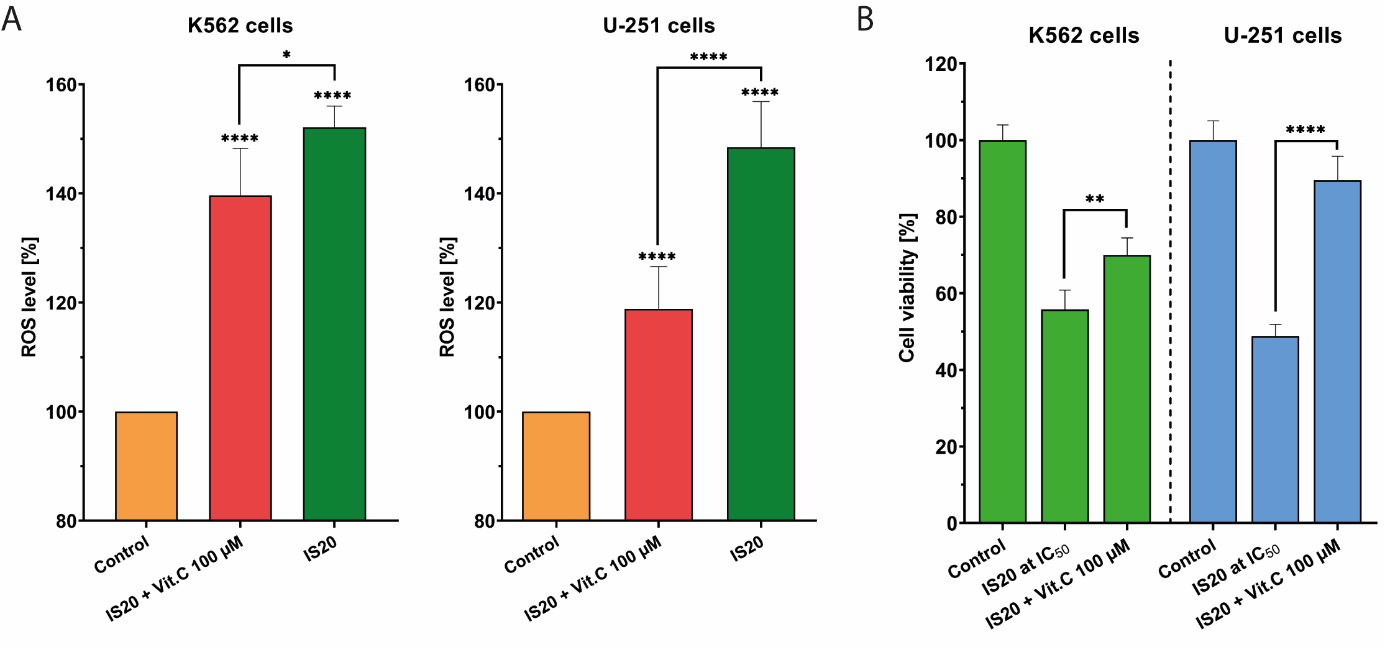


**Figure S1.** Effect of IS20 (IC_50_ concentration) and a combination IS20 with Vitamin C (100 uM) on reactive oxygen species (ROS) generation after 24 h (A) and cellular proliferation after 72 h (B) in U-251 and K562 cells. Statistical significance is presented above as *p<0.05, ****p<0.0001 relative to the respective control (untreated cells) and IS20 with Vitamin C, statistical analysis was done using one-way ANOVA with Bonferroni’s post-hoc test (n=3).


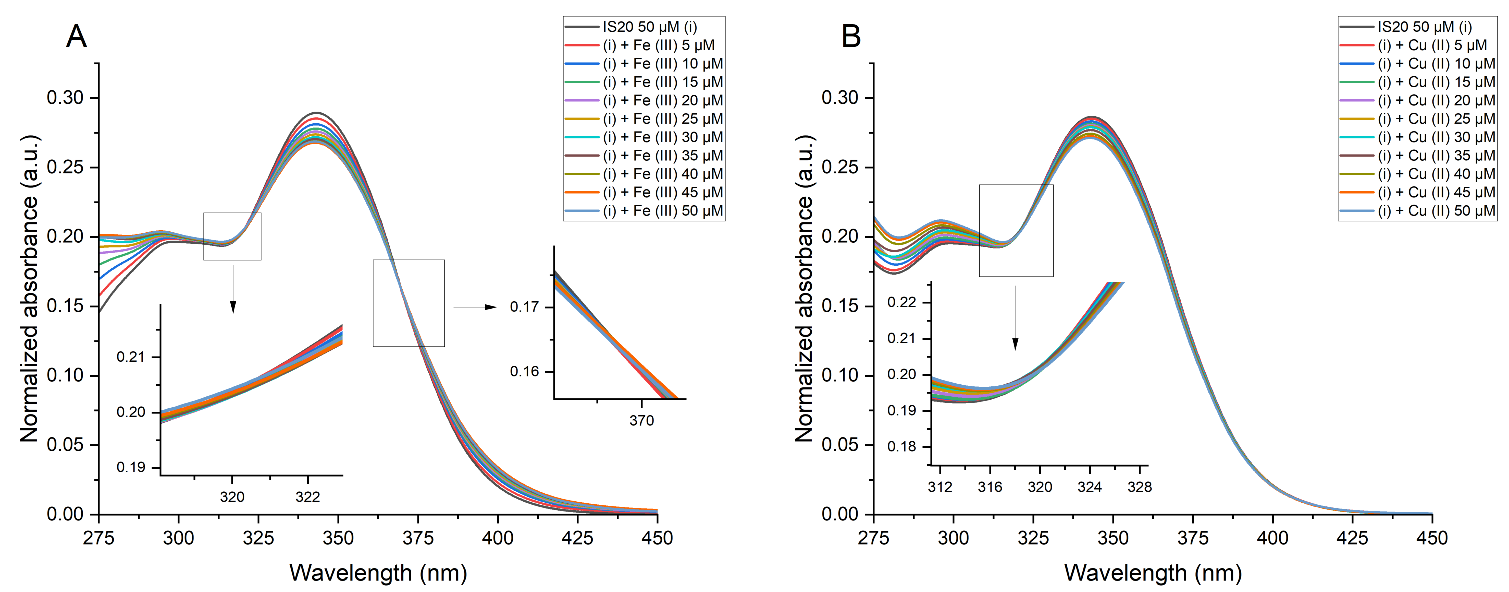


**Figure S2.** Spectroscopic titrations spectra of IS20 with Fe^3+^ (A) and Cu^2+^ (B) ions after normalization.

**
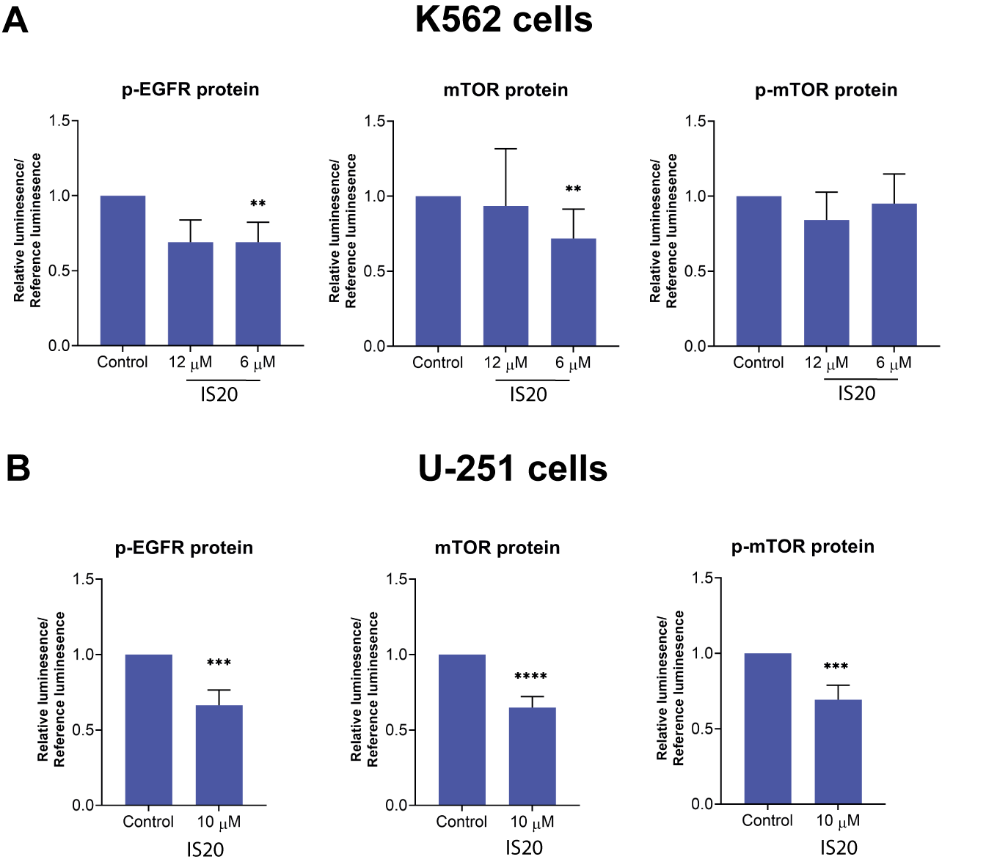
**

**Figure S3.** The expression levels of p-EGFR, mTOR and p-mTOR after exposure to IS20 in K562 (A) and U-251 (B) cells was measured using Lumit Immunoassay. Statistical significance is presented above as *p<0.05, **p<0.01, ***p<0.001, ****p<0.0001 relative to the respective control, statistical analysis was done using one-way ANOVA with Bonferroni’s post-hoc test.


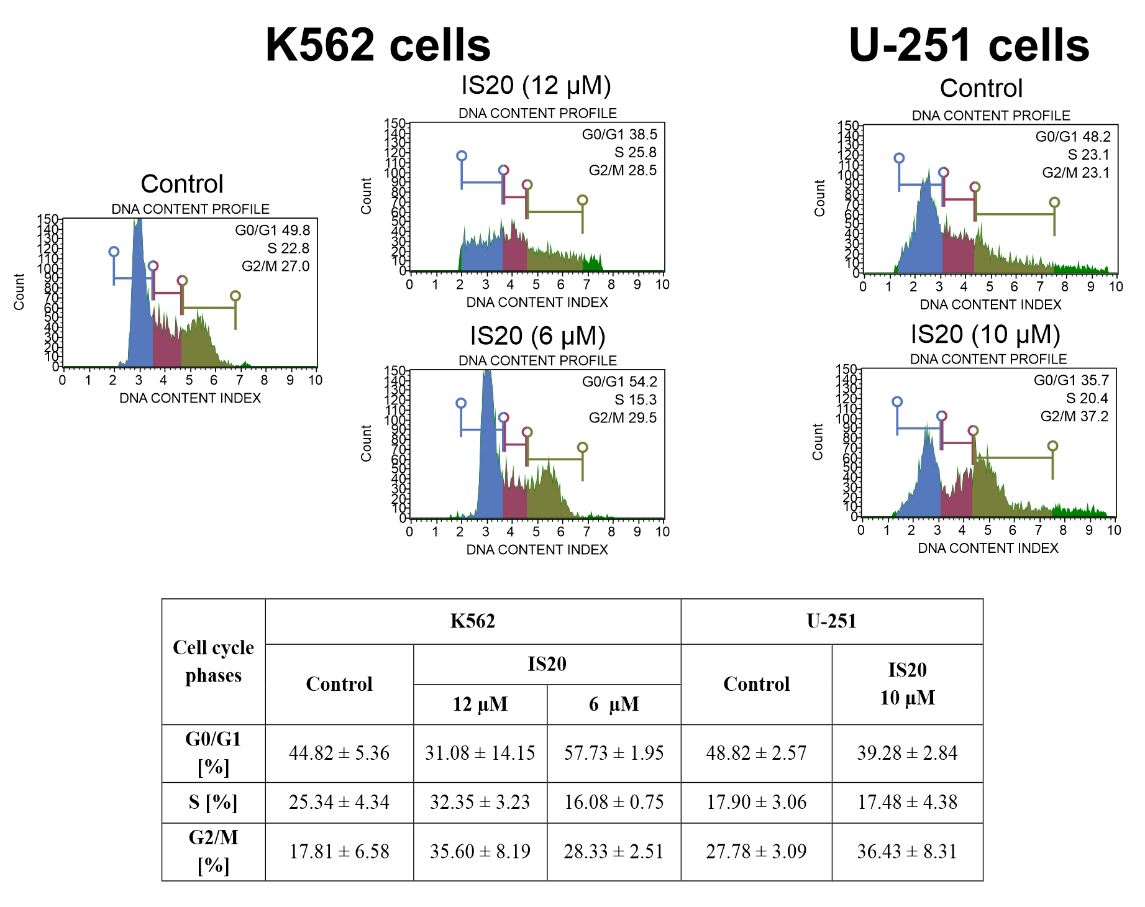


**Figure S4.** The influence of IS20 on the cell cycle arrest in the K562 and U-251 cells. The representative histograms present the results after a 24-hour incubation with IS20. The results of the with the percentage of cells in each phase of the cell cycle from all performed experiments are presented in the table as the mean ± SD.


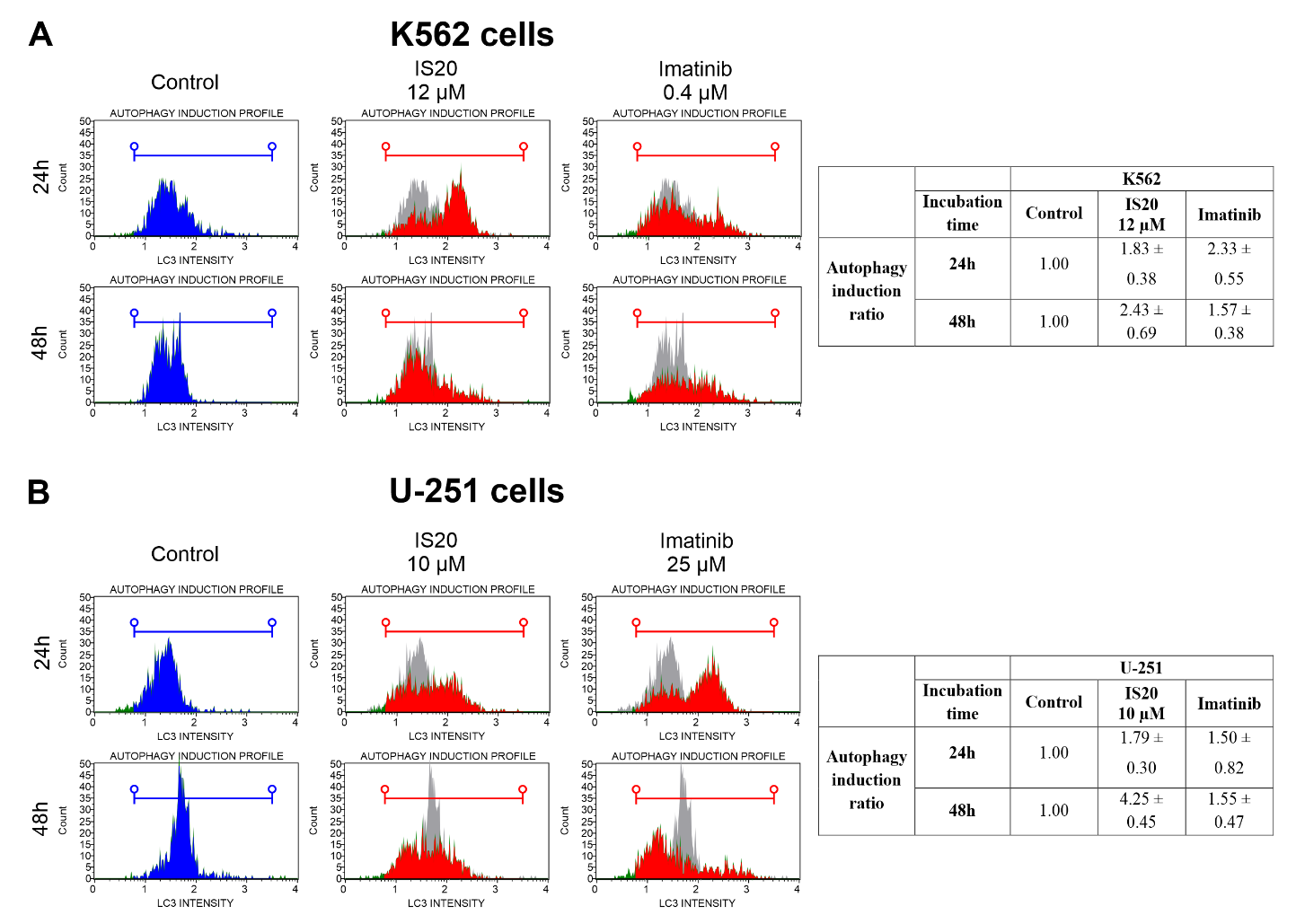


**Figure S5.** The influence of IS20 on the induction of autophagy in the K562 (A) and U-251 (B) cells. The representative histograms present the results after a 24- or 48-hour incubation with IS20 and imatinib. The results of the autophagy induction ratio from all performed experiments are presented in the table as the mean ± SD.

Images of the gels prepared during this study. All gels are uncropped and unmodified. Proteins relevant to this study are marked in boxes along with reference proteins.

A.

U-251 cells

PARP


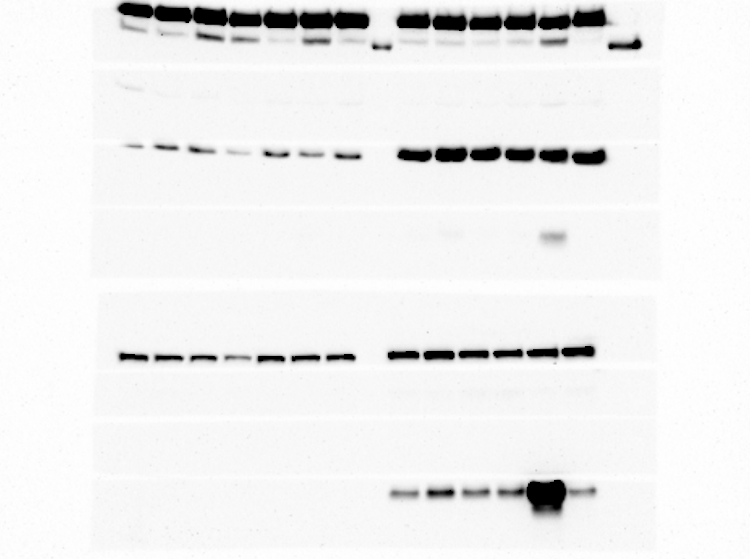


Different Experiment

IS20 10 µM

Control

IS20 10 µM

Control

Different Experiment

vinculin

HO-1

GAPDH

Gel with a molecular weight marker in CCD camera Merged images above

(colorimetric detection)

160 kDa

110 kDa

80 kDa

60 kDa

50 kDa

40 kDa

30 kDa

20 kDa


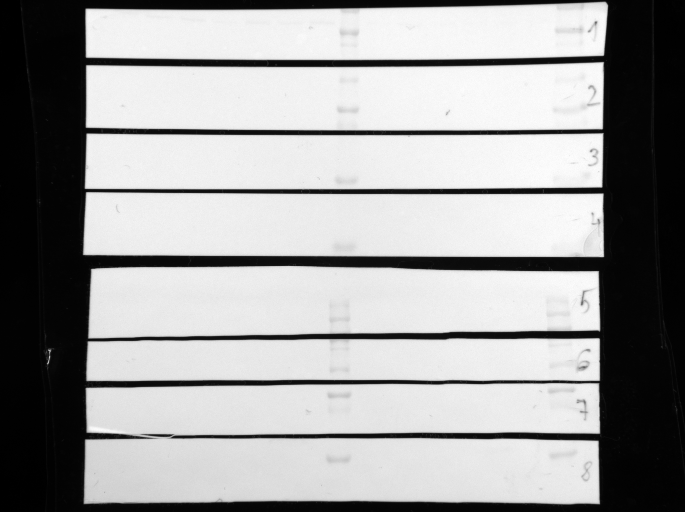

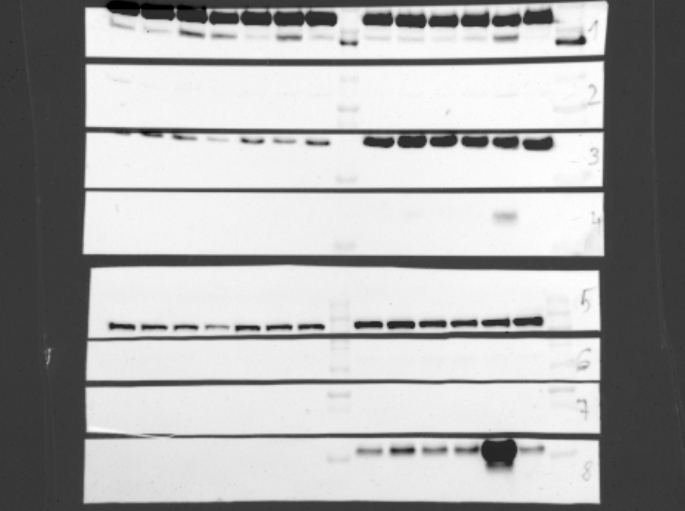


260 kDa

160 kDa

110 kDa

80 kDa

60 kDa

50 kDa

40 kDa

30 kDa

B.

U-251 cells


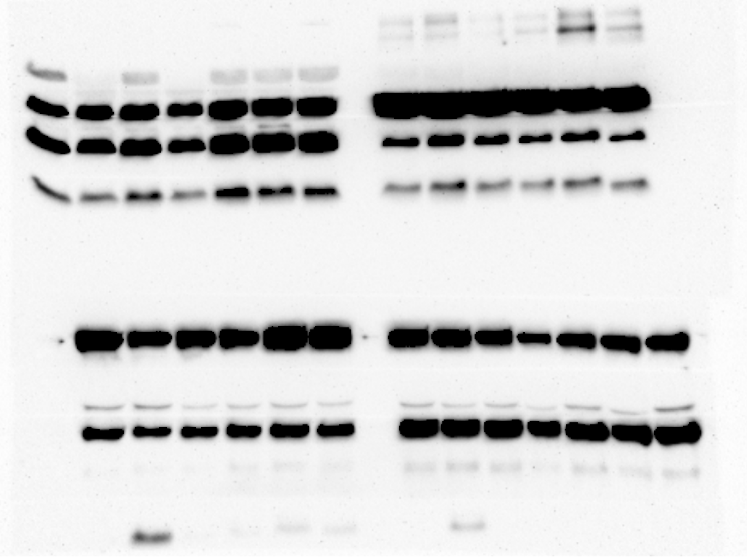


IS20 10 µM

Control

IS20 10 µM

Control

Different Experiment

Different Experiment

IS20 10 µM

Control

vinculin

β-actin

BID

p21

U-251 cells

Gel with a molecular weight marker in CCD camera Merged images above

(colorimetric detection)

160 kDa

110 kDa

80 kDa

60 kDa

40 kDa

20 kDa

15 kDa


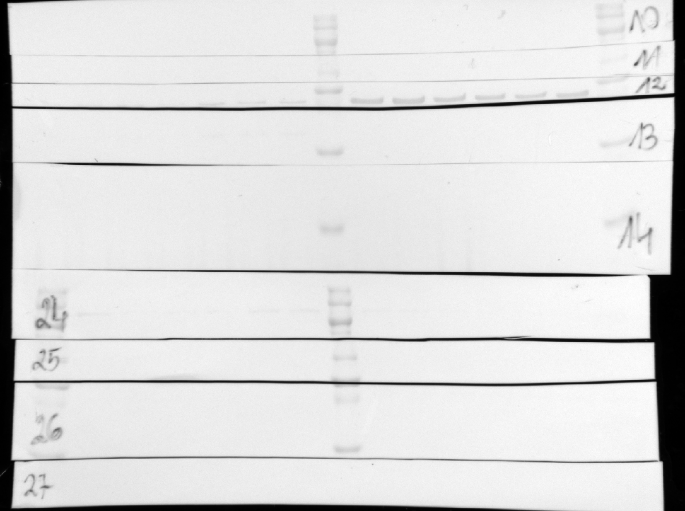

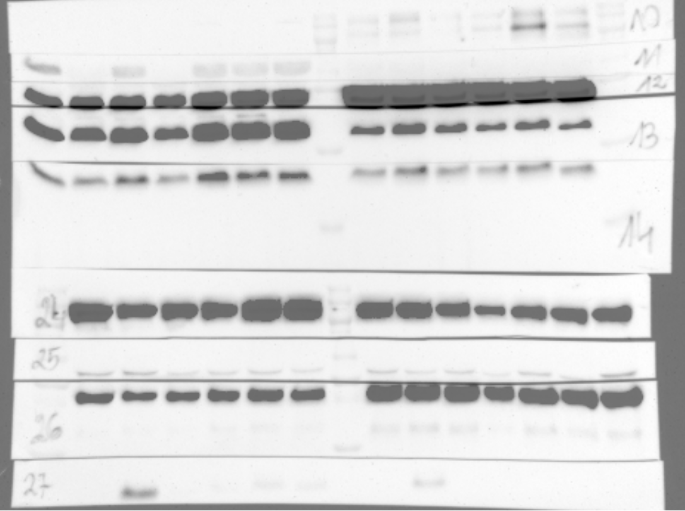


260 kDa

160 kDa

110 kDa

80 kDa

60 kDa

50 kDa

40 kDa

30 kDa

U-251 cells

(longer exposure)


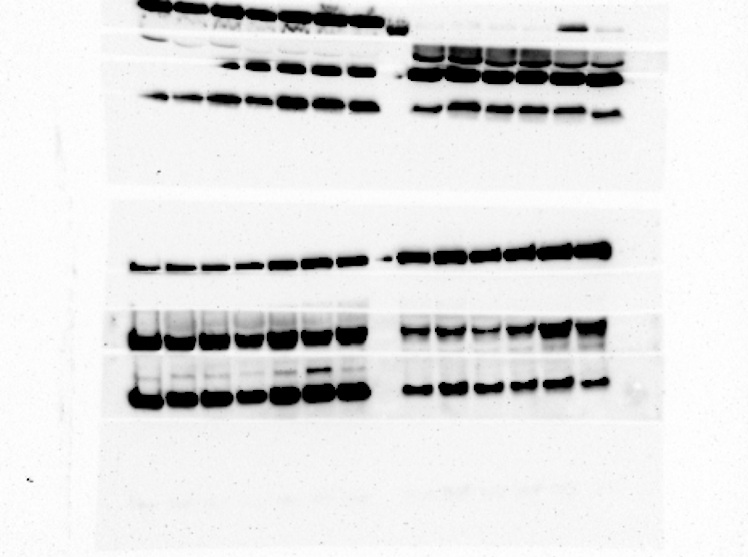
C.

U-251 cells

HIF-1α


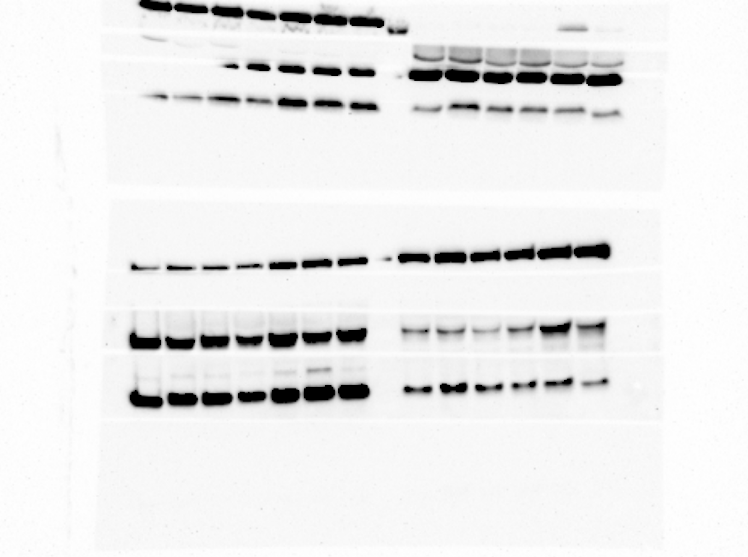


Different Experiment

IS20 10 µM

Control

Different Experiment

Different Experiment

IS20 10 µM

Control

IS20 10 µM

Control

cyclin E1

HIF-1α

GAPDH

cdc2

vinculin

GAPDH

Gel with a molecular weight marker in CCD camera Merged images above

(colorimetric detection)

160 kDa

110 kDa

60 kDa

40 kDa

30 kDa

20 kDa

50 kDa


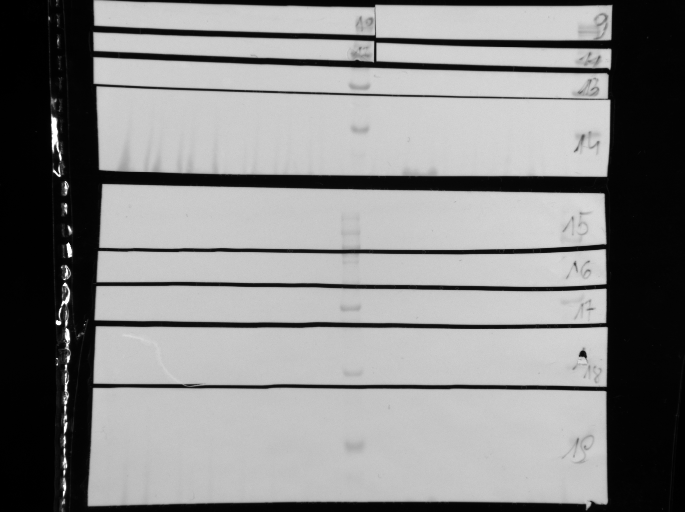

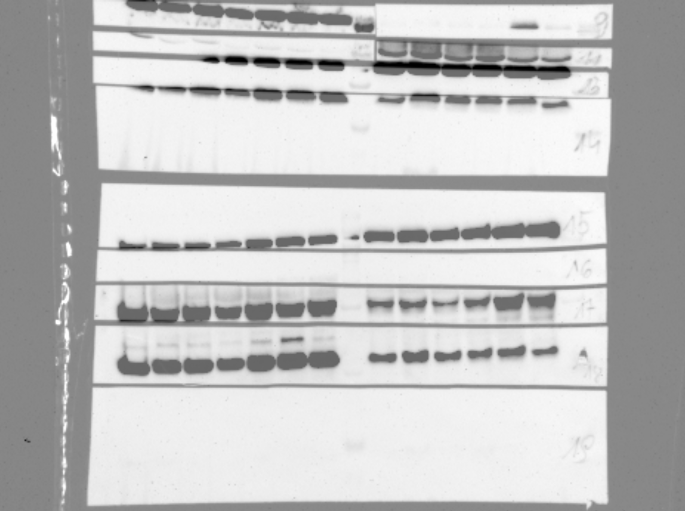


260 kDa

160 kDa

110 kDa

80 kDa

60 kDa

50 kDa

40 kDa

30 kDa

20 kDa

D.

U-251 cells


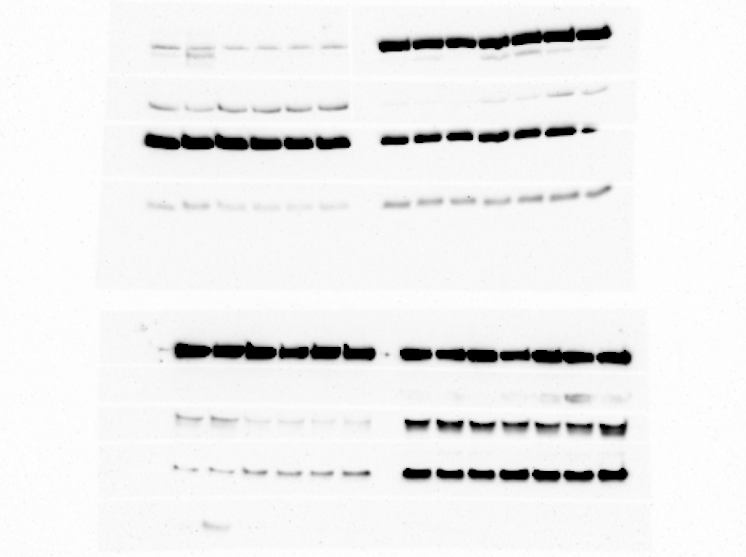


Different Experiment

Different Experiment

IS20 12 µM

Control

IS20 6 µM

cdc2

vinculin

IS20 10 µM

Control

Different Experiment

p53

GAPDH

K562 cells

Gel with a molecular weight marker in CCD camera Merged images above

(colorimetric detection)


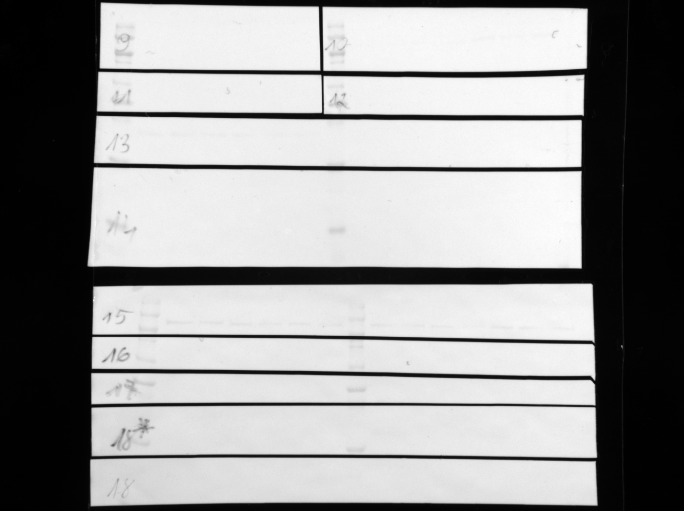

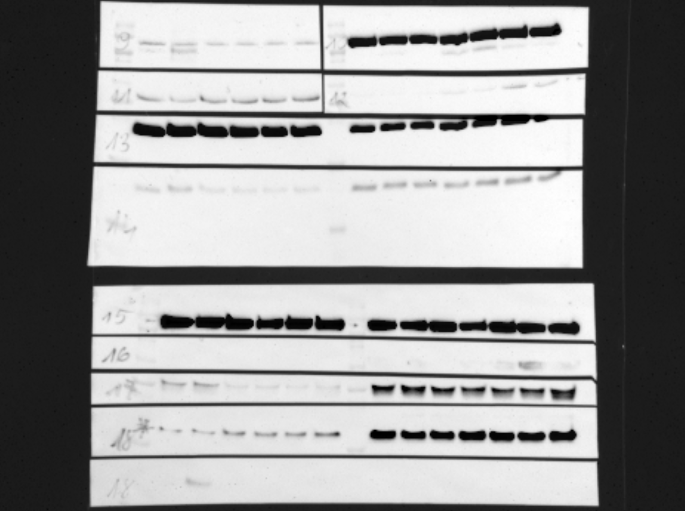


260 kDa

160 kDa

110 kDa

80 kDa

60 kDa

50 kDa

40 kDa

30 kDa

260 kDa

160 kDa

110 kDa

80 kDa

60 kDa

50 kDa

40 kDa

30 kDa

20 kDa

E.

K562 cells

U-251 cells


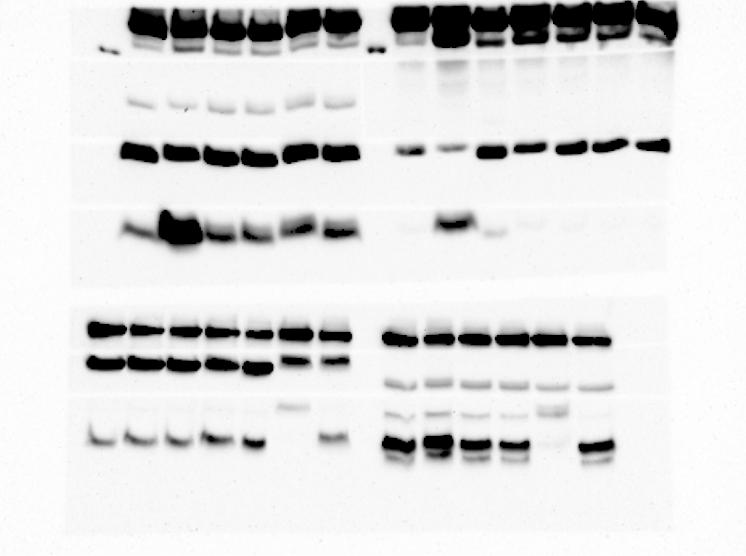


IS20 12 µM

Control

IS20 6 µM

IS20 12 µM

Control

IS20 6 µM

Different Experiment

Different Experiment

Different Experiment

Different Experiment

IS20 10 µM

Control

vinculin

cathepsin b

vinculin

K562 cells

U-251 cells

cathepsin b

PARP

GAPDH

Gel with a molecular weight marker in CCD camera Merged images above

(colorimetric detection)

160 kDa

110 kDa

60 kDa

40 kDa

30 kDa

20 kDa

50 kDa

80 kDa


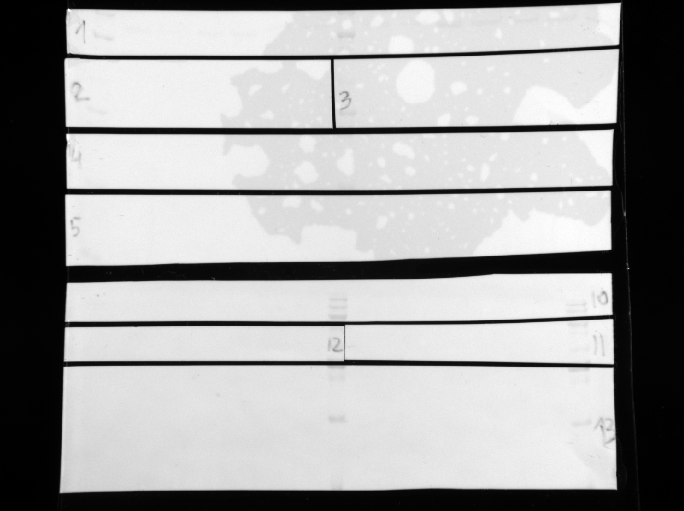

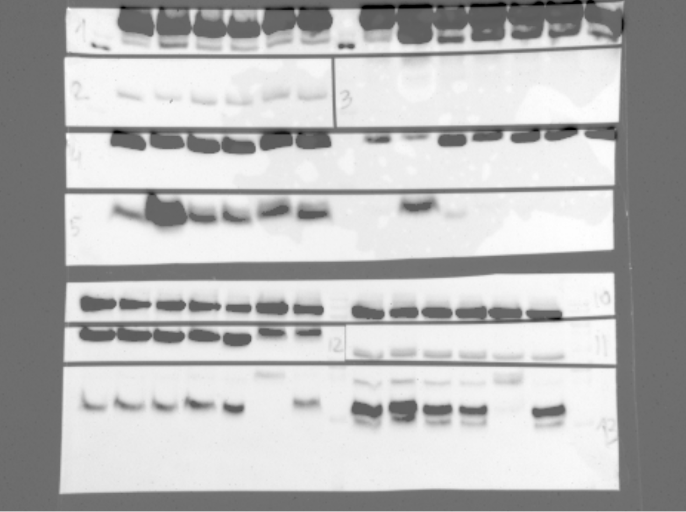


260 kDa

160 kDa

110 kDa

80 kDa

60 kDa

50 kDa

40 kDa

30 kDa

20 kDa


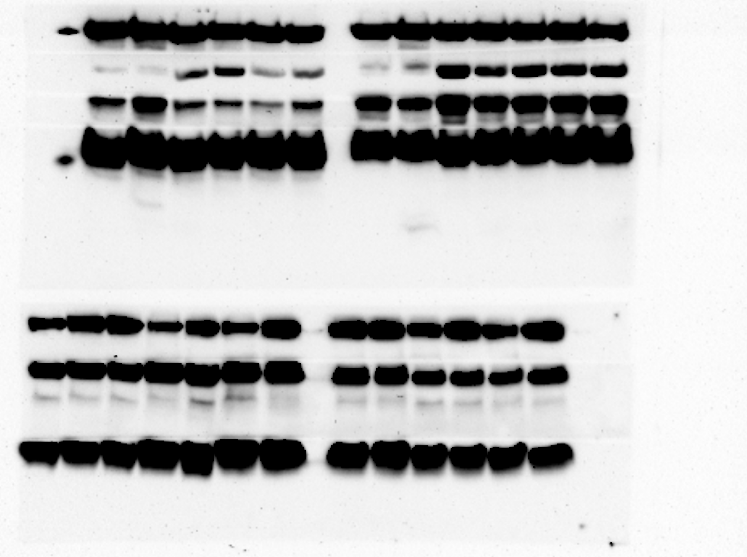
F.

vinculin

K562 cells


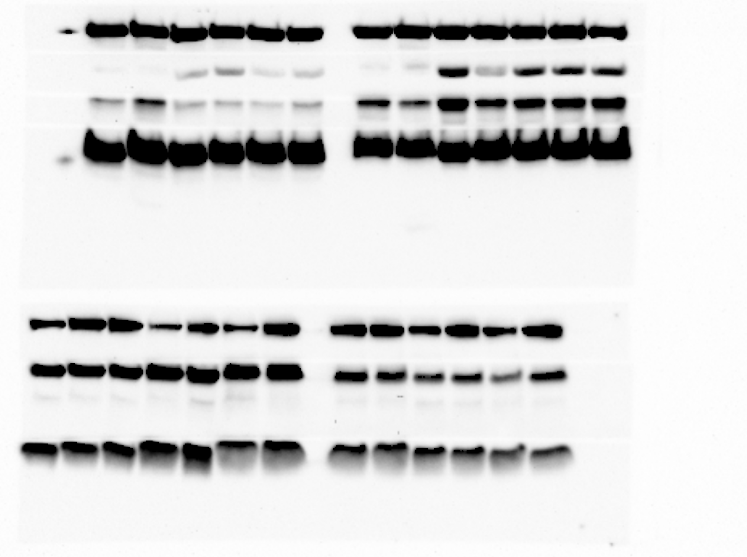


IS20 12 µM

Control

IS20 6 µM

Different Experiment

Different Experiment

Different Experiment

IS20 12 µM

Control

IS20 6 µM

K562 cells (longer exposure)

cyclin E1

AIF

vinculin

caspase-9

BID

caspase-9

vinculin

K562 cells

Gel with a molecular weight marker in CCD camera Merged images above

(colorimetric detection)

260 kDa

160 kDa

110 kDa

80 kDa

60 kDa

50 kDa

40 kDa

30 kDa

15 kDa


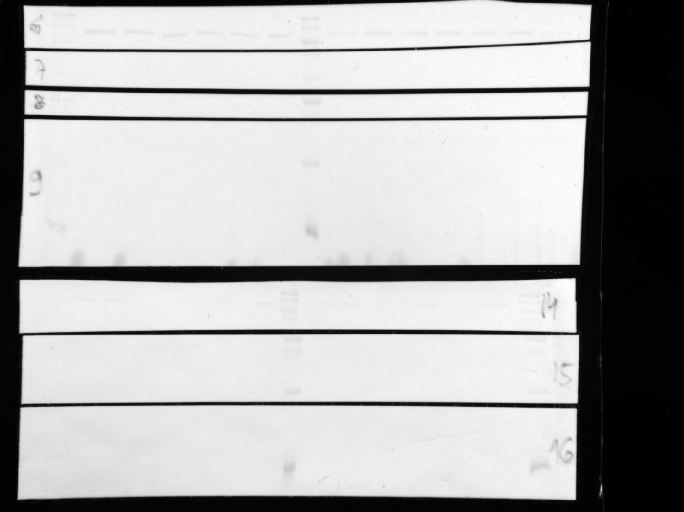

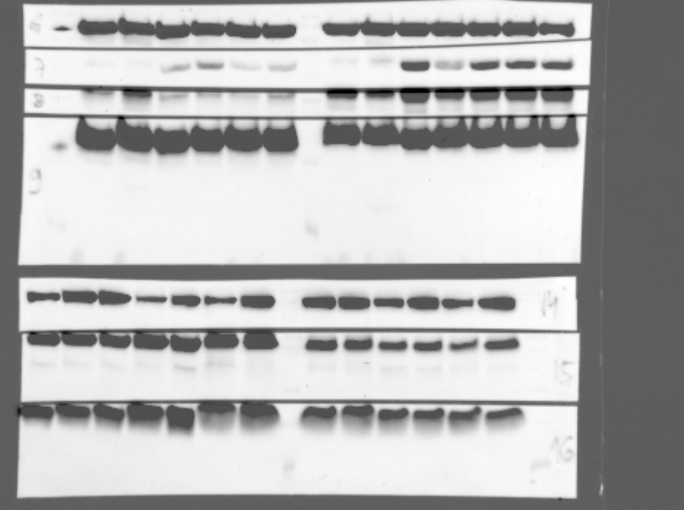


260 kDa

160 kDa

110 kDa

80 kDa

60 kDa

50 kDa

40 kDa

30 kDa

15 kDa

20 kDa

G.


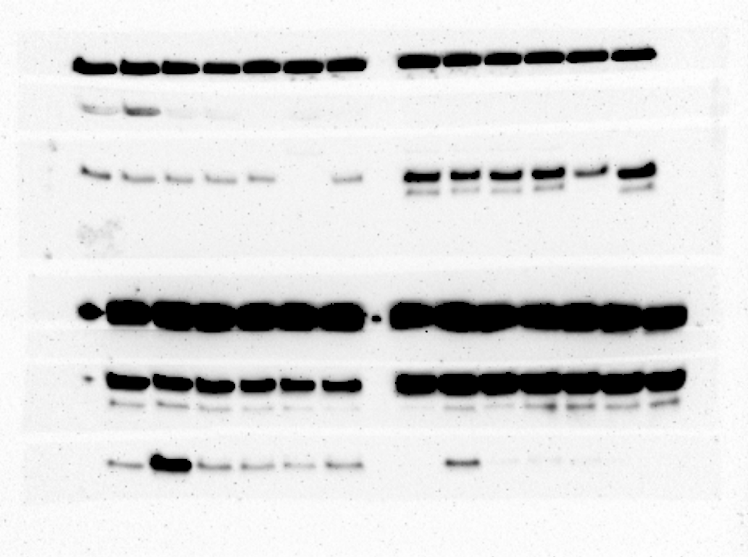


Different Experiment

Different Experiment

Different Experiment

IS20 12 µM

Control

IS20 6 µM

K562 cells

vinculin

p21

Gel with a molecular weight marker in CCD camera Merged images above

(colorimetric detection)


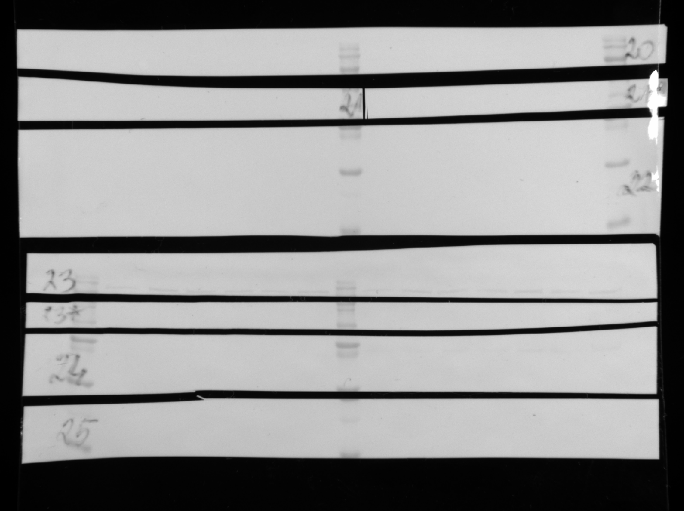

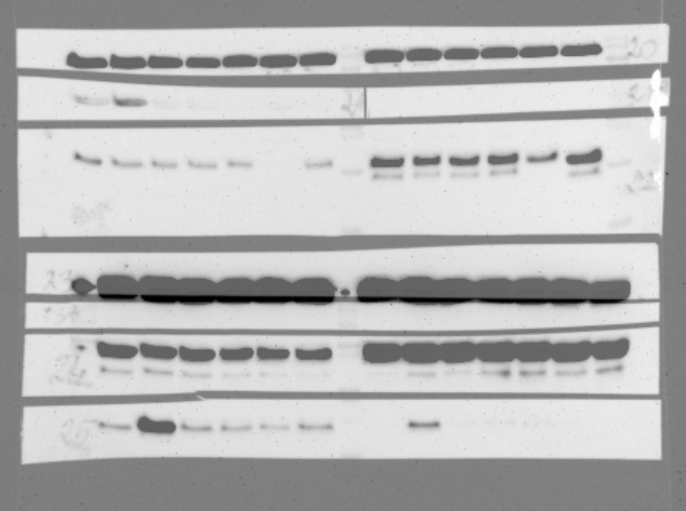


260 kDa

160 kDa

110 kDa

40 kDa

30 kDa

20 kDa

10 kDa

15 kDa

H.


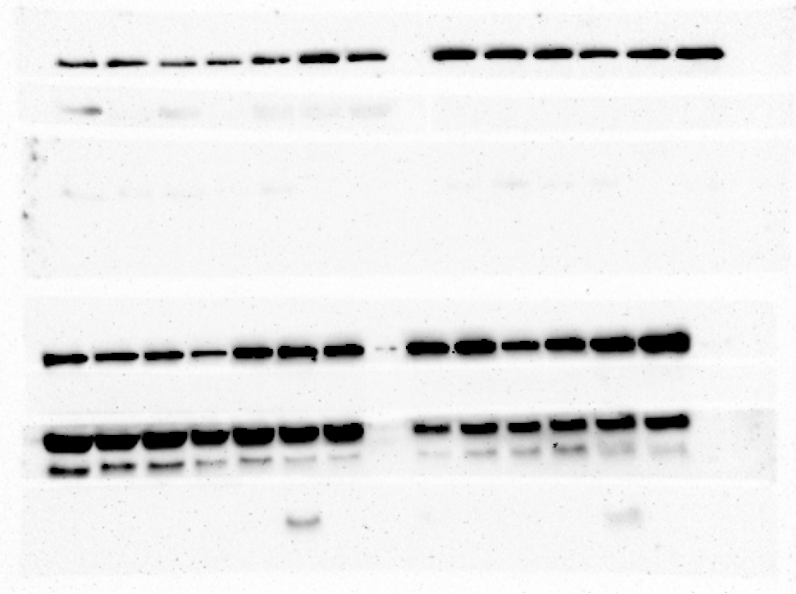


Different Experiment

IS20 10 µM

Control

U-251 cells

caspase-9

vinculin

Gel with a molecular weight marker in CCD camera Merged images above

(colorimetric detection)


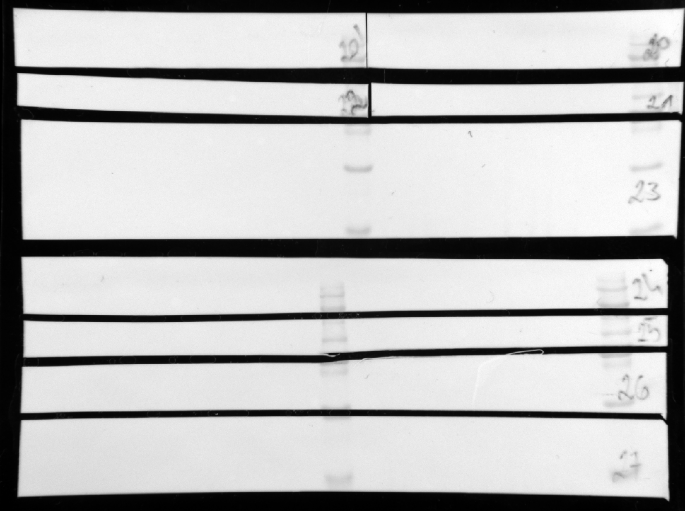

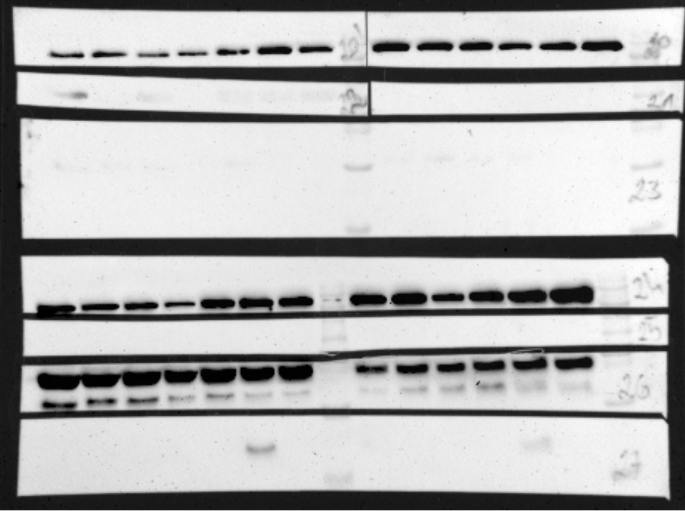


260 kDa

160 kDa

110 kDa

80 kDa

60 kDa

50 kDa

40 kDa

30 kDa

20 kDa

I.

U251 cells

(longer exposure)

K562 cells

K562 cells

(longer exposure)

U251 cells


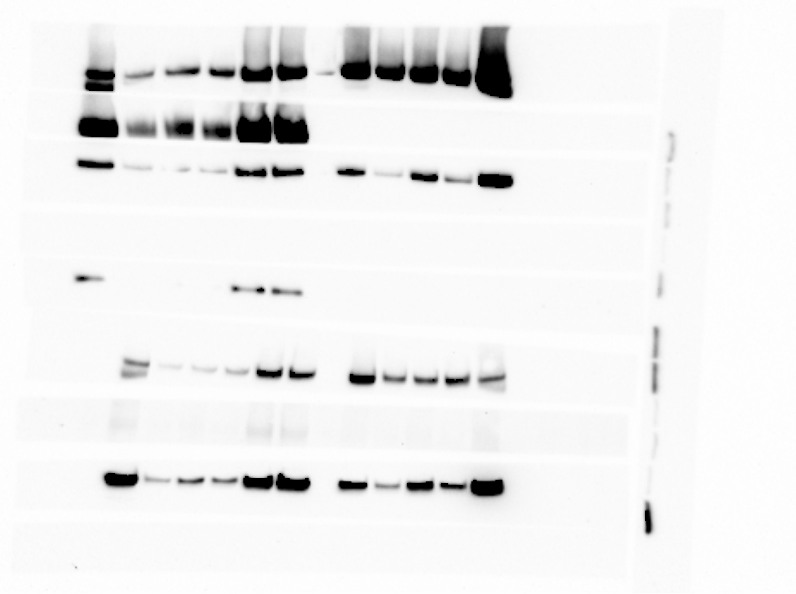

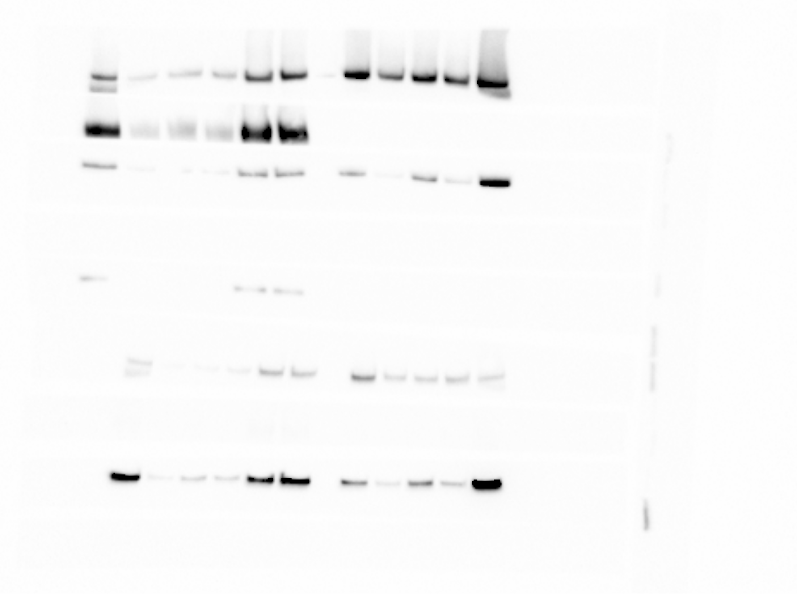


Different Experiment

Different Experiment

Different Experiment

Different Experiment

IS20 12 µM

Control

IS20 6 µM

IS20 10 µM

Control

p-AKT

vinculin

vinculin

p-mTOR

mTOR

K562 cells

(longer exposure)


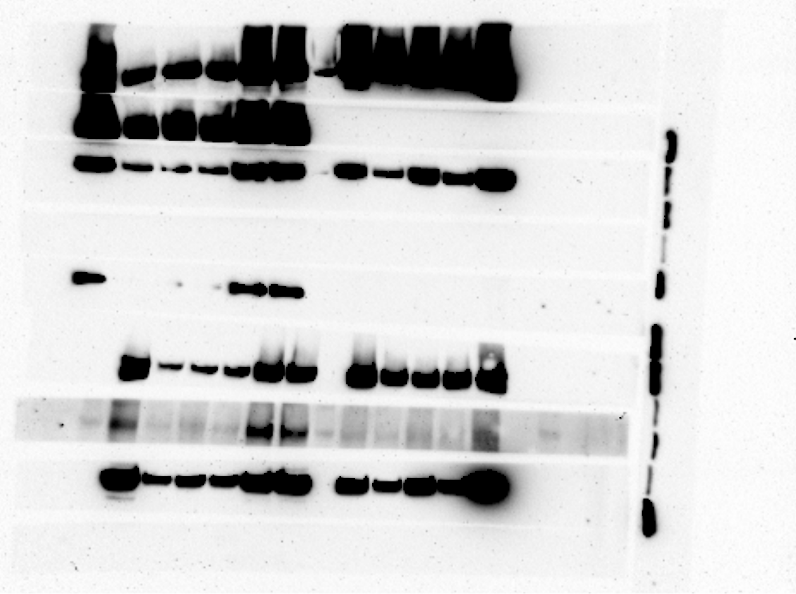


Different Experiment

IS20 12 µM

Control

IS20 6 µM

vinculin

p-EGFR

Gel with a molecular weight marker in CCD camera Merged images above


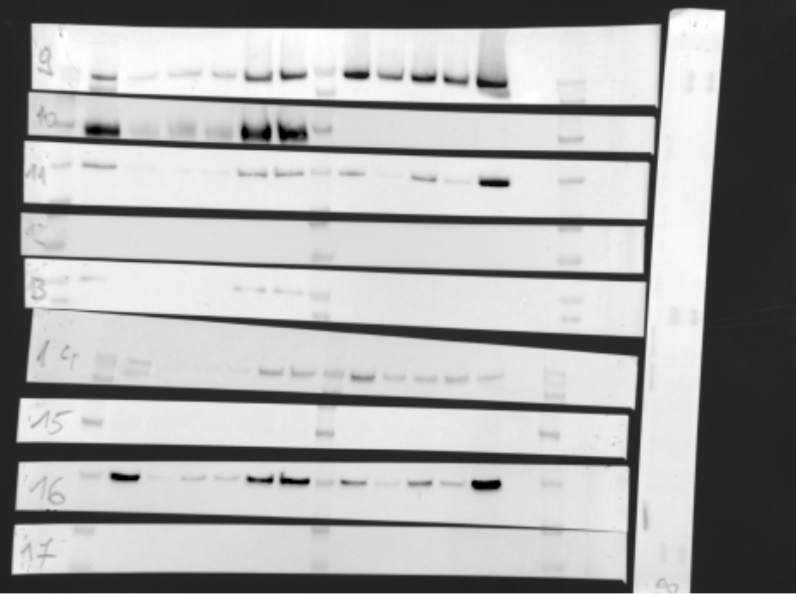

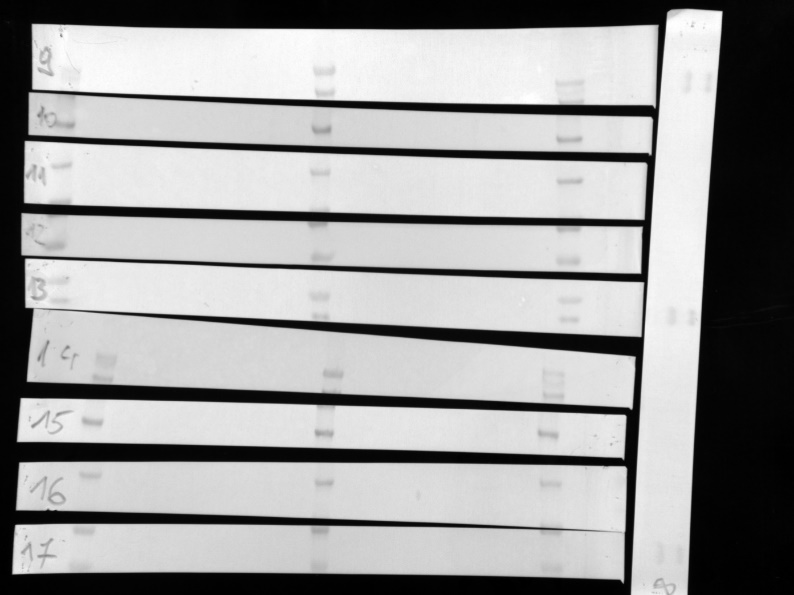


40 kDa

50 kDa

300 kDa

250 kDa

180 kDa

130 kDa

100 kDa

70 kDa

300 kDa

250 kDa

180 kDa

130 kDa

100 kDa

70 kDa

50 kDa

40 kDa

**
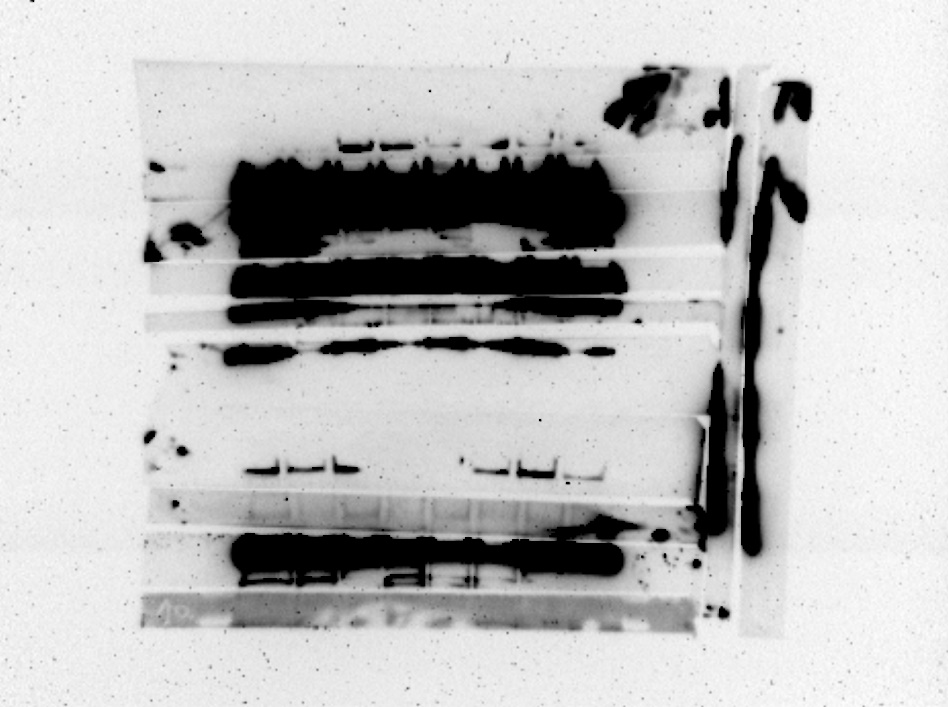
**J.

U251 cells

U251 cells (longer exposure)

**
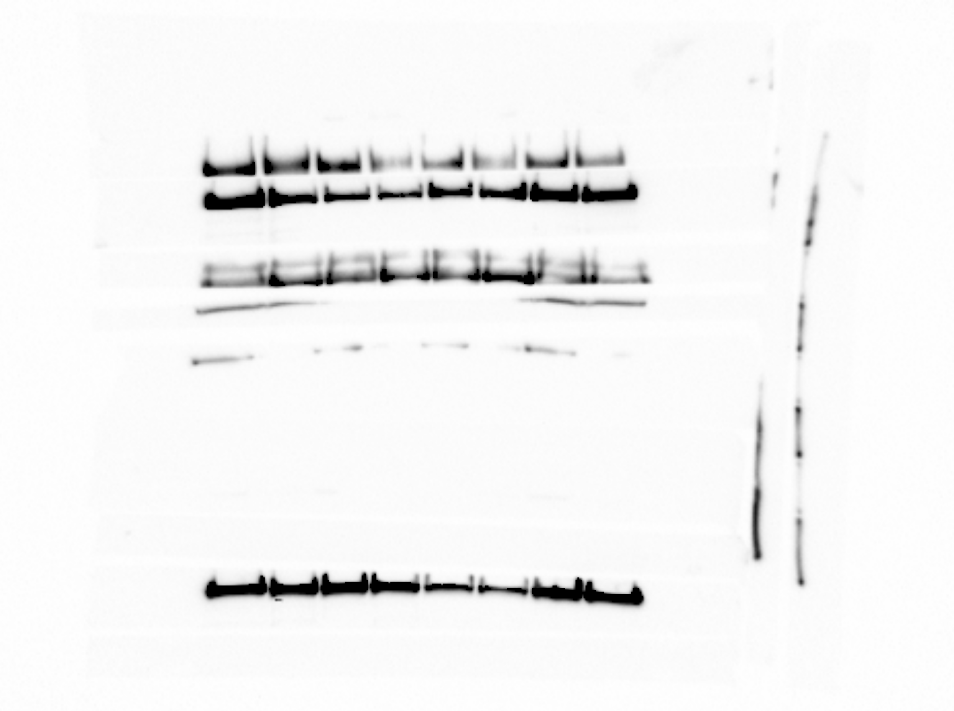
**

IS20 10 µM

Control

Different Experiment

Different Experiment

IS20 10 µM

Control

p-ERK1/2

p-EGFR

vinculin

vinculin

EGFR

Gel with a molecular weight marker in CCD camera Merged images above

**
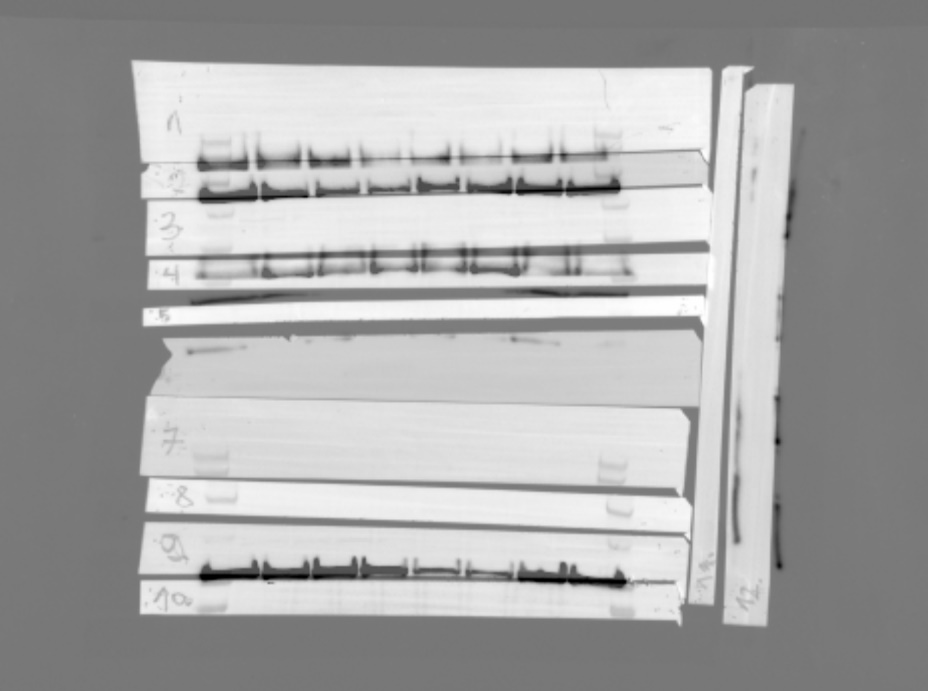
**(colorimetric detection)

300 kDa

250 kDa

180 kDa

130 kDa

100 kDa

70 kDa

50 kDa

40 kDa

**
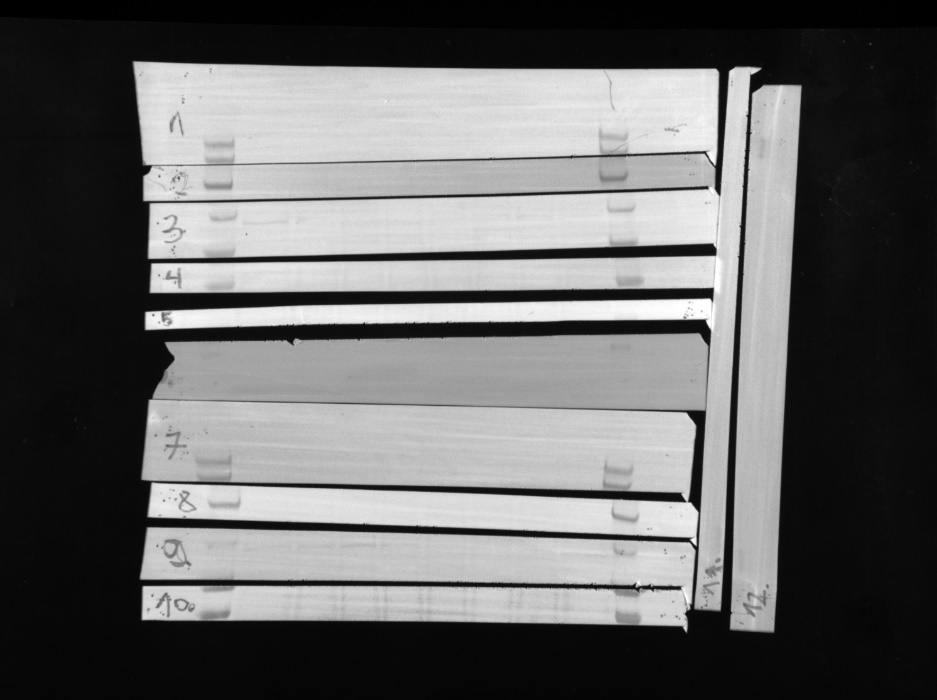
**

300 kDa

250 kDa

180 kDa

130 kDa

100 kDa

70 kDa

50 kDa

40 kDa

K.


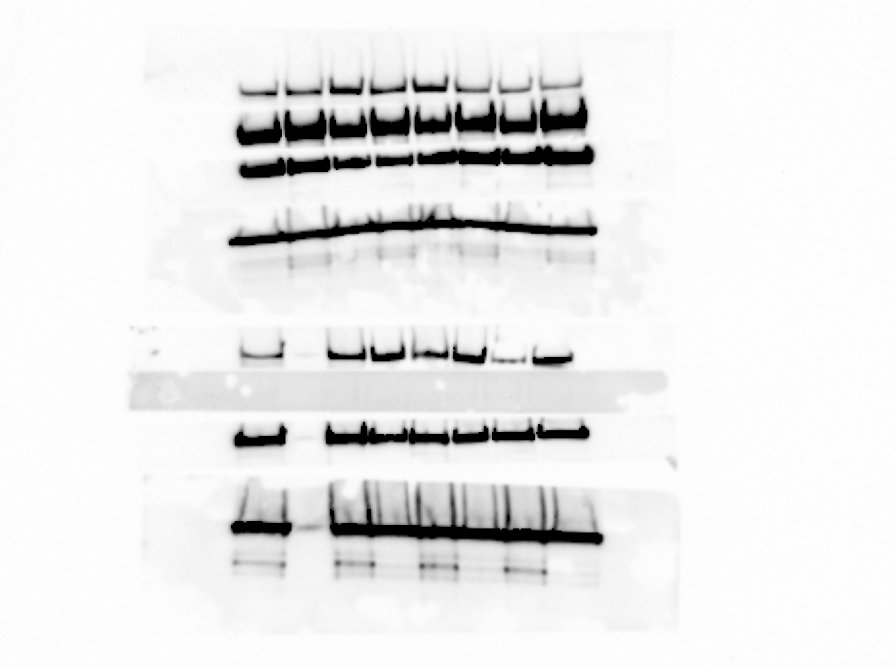


Different Experiment

IS20 10 µM

Control

U251 cells

vinculin

p-mTOR

Gel with a molecular weight marker in CCD camera Merged images above

(colorimetric detection)


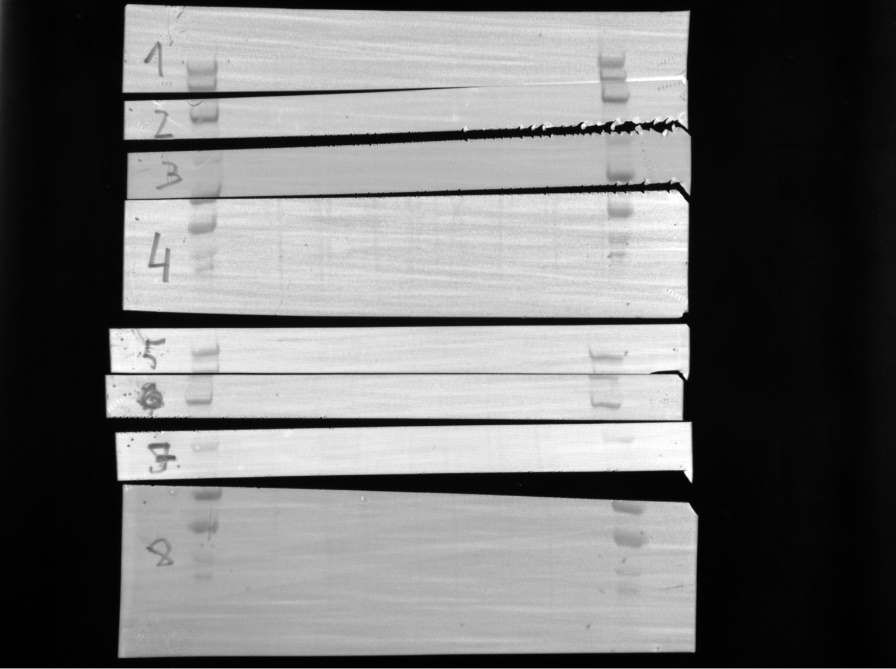

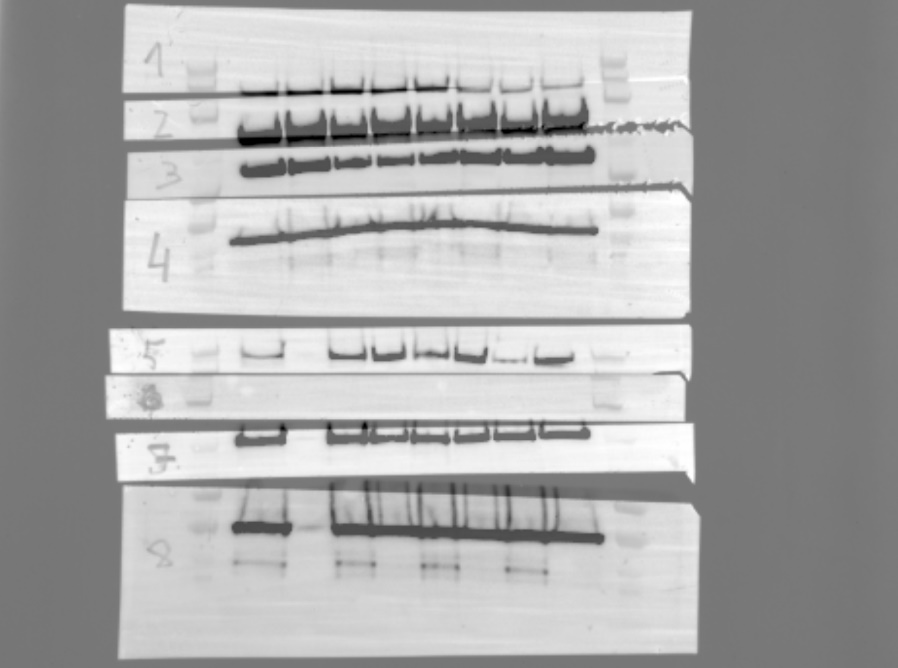


300 kDa

250 kDa

180 kDa

130 kDa

100 kDa

70 kDa

50 kDa

40 kDa

**Figure S6.** The uncropped Western blots (**A**-**K**).
